# A stacking ensemble system for identifying the presence of histological variants in bladder carcinoma: a multicenter study

**DOI:** 10.3389/fonc.2024.1469427

**Published:** 2025-01-10

**Authors:** Canjie Peng, Quanhao He, Fajin Lv, Qing Jiang, Yong Chen, Zongjie Wei, Yingjie Xv, Fangtong Liao, Mingzhao Xiao

**Affiliations:** ^1^ Department of Urology, The First Affiliated Hospital of Chongqing Medical University, Chongqing, China; ^2^ Department of Radiology, The First Affiliated Hospital of Chongqing Medical University, Chongqing, China; ^3^ Department of Urology, The Second Affiliated Hospital of Chongqing Medical University, Chongqing, China; ^4^ Department of Urology, Chongqing University Fuling Hospital, Chongqing, China

**Keywords:** machine learning, bladder cancer, histological variants, radiomics, artificial intelligence

## Abstract

**Purpose:**

To create a system to enable the identification of histological variants of bladder cancer in a simple, efficient, and noninvasive manner.

**Material and methods:**

In this multicenter diagnostic study, we retrospectively collected basic information and CT images about the patients concerned from three hospitals. An interactive deep learning-based bladder cancer image segmentation framework was constructed using the Swin UNETR algorithm for further features extraction. Radiomic features and deep learning features were extracted for further stacking ensemble system construction. The segmentation model’ performance was assessed by using Dice Similarity (Dice) metrics, Intersection Over Union (IOU), Sensitivity (SEN) and Specificity (SPE). To evaluate the system’s performance, we used the Receiver Operating Characteristics (ROC) curve, the Accuracy Score (ACC) and Decision Curve Analysis (DCA).

**Results:**

410 patients from one hospital were included in the training set, while 60 patients from two other hospitals were included in the test set. A total of 50 features comprising 46 radiomic features and 4 deep learning features were finally retained for further stacking ensemble model building. The interactive segmentation model and system exhibited excellent performance in both training (Dice = 0.78, IOU = 0.65, SEN = 0.83, SPE = 1.00, AUC = 0.940, ACC = 0.868) and testing datasets (Dice = 0.80, IOU = 0.67, SEN = 0.89, SPE = 1.00, AUC = 0.905, ACC = 0.900).

**Conclusion:**

We successfully constructed a stacking ensemble machine learning model for early, non-invasive identification of histological variants in bladder cancer which will help urologists make clinical decisions.

## Backgrounds

Bladder cancer is one of the most common malignant tumors in the urinary system globally, and its incidence is gradually increasing (1). Approximately 75% of the bladder cancers cases were classified as pure urothelial carcinoma (PUC), while the remaining 25% present histological variants (2, 3), such as squamous differentiation, glandular differentiation, Micropapillary urothelial carcinoma. As manifested by the WHO 2022 classification of urothelial tumors of the urinary tract, this classification presented a subtype of invasive urothelial carcinoma with histological variants and divergent differentiation encompassing tumors that exhibit a mix of “typical type” urothelial carcinoma and other morphologies (4, 5). Correct understanding of bladder cancer’s pathological types and appropriate treatment is crucial for improving the prognosis of patients (6, 7). For the vast majority of patients with bladder cancer (pure urothelial carcinoma, PUC) scheduled for radical cystectomy, cisplatin-based neoadjuvant therapy has proven significantly effective in enhancing patients’ postoperative prognosis (8, 9). However, in the minority of patients with bladder cancer in the presence of histological variants, they are more prone to early lymph node metastasis and generally have a poorer prognosis (4, 10). Additionally, they often respond poorly to cisplatin-based neoadjuvant chemotherapy (11). Therefore, early identification and tailored treatment adjustments for these patients are extremely vital to improve their prognosis.

The current gold standard for diagnosing the type of pathology in bladder cancer patients relies on the pathologist’s correct identification of surgical or biopsy specimens (12, 13). However, the whole process suffers from the following challenges: (1) All tumor tissue after radical cystectomy can be accurately identified by the pathologist and evaluated for pathological types associated with histological variants (since the pathological specimen is the entire bladder), meanwhile, the vast majority of pathological specimens are obtained by transurethral cystoscopic biopsy or transurethral resection of bladder tumor (TURBT); This often results in residual tumor tissue remaining in the patient’s bladder. If there is histologically variant tumor tissue remaining in the patient that was not retrieved in the biopsy, the pathologist will make an incorrect initial determination of the pathologic type of bladder cancer. This misclassification can subsequently impact the urologist’s treatment decisions (14, 15). (2) Considering that survival rate and prognosis of bladder cancer patients with histological variants are worse, delays in obtaining biopsy or TURBT results and subsequent pathology analysis can lead to disease progression and worsen the patient’s condition. (3) Meanwhile, both biopsy and TURBT are invasive operations, posing additional risks and discomfort for the patient. Based on this, there is an urgent need to develop a simple, efficient, and noninvasive method for early identification of patients with bladder cancer combined with histologic variants.

Artificial intelligence has made major advances in the field of medicine in recent years and is poised to revolutionize the practice of medicine (16, 17). The most significant advantage of AI over clinicians is the ability to effectively utilize various medical data to assist clinicians in disease diagnosis, prognosis (18–21). In the field of bladder cancer, previous researches have indicated that it is feasible to apply radiomics and machine learning to access pathological grade and muscle invasiveness in bladder carcinoma (22–25). However, few studies have been conducted to identify histological variants of bladder cancer by radiomics, especially CT. And we posit that the application of radiomics combined with machine learning could also effectively predict histological variants in bladder cancer.

Therefore, we created a system to enable the identification of histological variants of bladder cancer in a simple, efficient, and noninvasive manner (the dependent variable (histological variant) of the classification task is a binary categorical variable, which can be divided into non-histological variant and histological variant). This will be used to guide the clinical practice of urologists, facilitating the selection of the most suitable treatment for patients.

## Materials and methods

### Patients

**Figure 1:**
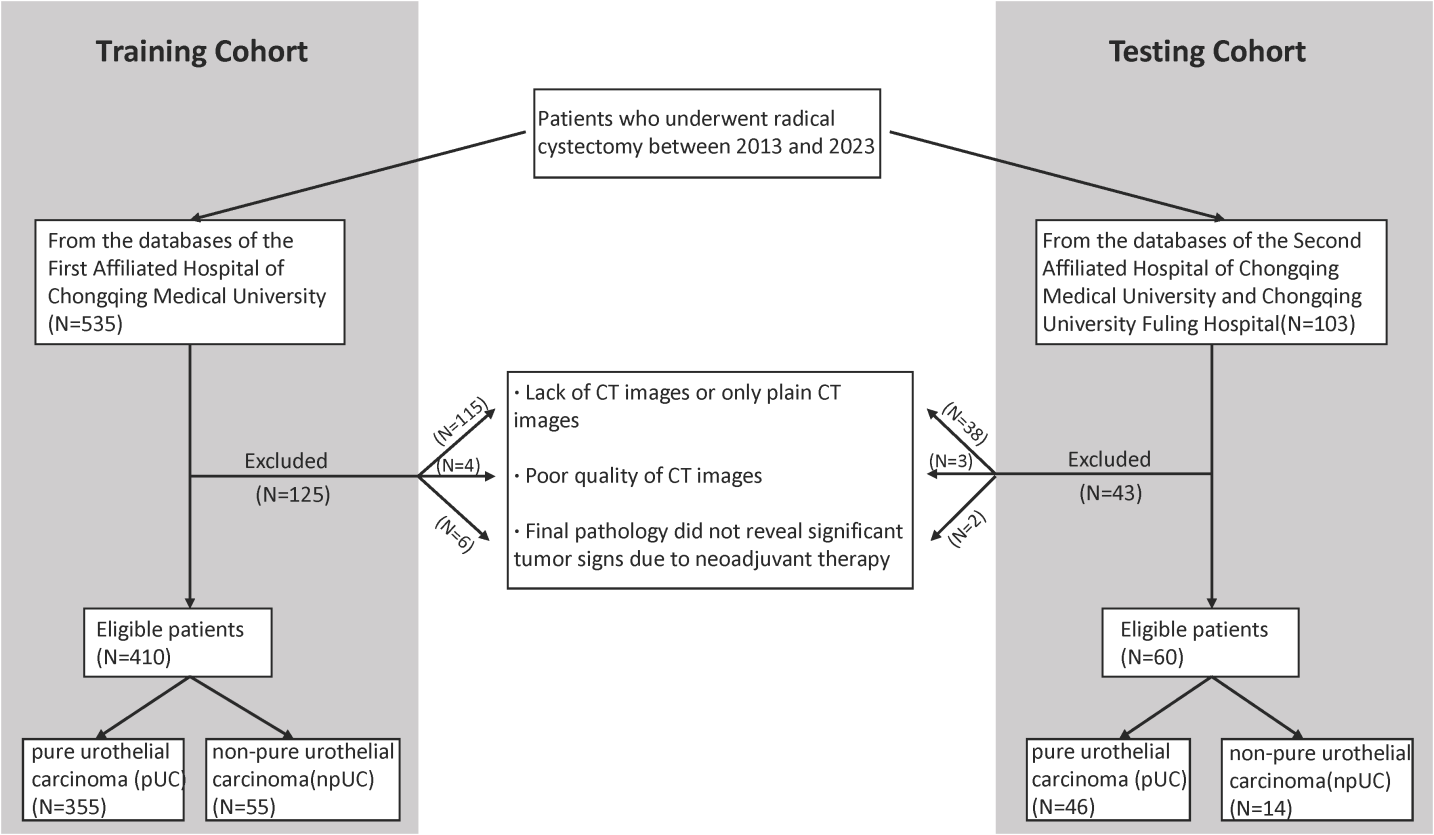
Flowchart shows patient selection.

We retrospectively gathered basic information and CT images of patients who underwent radical cystectomy between 2013 and 2023 from the databases of three completely independent healthcare institutions, the First Affiliated Hospital of Chongqing Medical University, the Second Affiliated Hospital of Chongqing Medical University, and Chongqing University Fuling Hospital. The exclusion criteria for this study were: (1) absence of CT images or availability of only plain CT images; (2) poor quality of CT images; and (3) patients whose final pathology reports did not reveal significant tumor signs due to neoadjuvant therapy. From the database of the First Affiliated Hospital of Chongqing Medical University, A total of 410 patients were finally included, including 55 patients with non-pure urothelial carcinoma (NPUC). From the databases of the Second Affiliated Hospital of Chongqing Medical University and Chongqing University Fuling Hospital, a total of 60 patients were finally included, including 14 patients with NPUC. A detailed distribution of basic information and pathology type of the included patient cohort across these hospitals is provided in **Table 1**. The postoperative pathological results of the participants will be used as the gold standard to assess whether the bladder cancer is associated with histological variation. The detailed data collection and screening process is shown in **Figure 1**. Data from the First Affiliated Hospital of Chongqing Medical University will be used for the model construction, while data from the Second Affiliated Hospital of Chongqing Medical University and Chongqing University Fuling Hospital will be used for the external validation which effectively avoided potential statistical dependence.

**Table 1:** A detailed distribution of basic information and pathology type of the included patient cohort across these hospitals.

Cohort	Hospital	Patients	Median Age(IQR)	Male/Female	Pathological Type	Number
Training cohort	The First Affiliated Hospital of Chongqing Medical University	410	66(60-74)	364/46	Pure urothelial carcinoma (PUC) UC with squamous differentiation UC with glandular differentiation Micropapillary UC Plasmacytoid UC Sarcomatoid UC Poorly differentiated UC Neuroendocrine lineage UC	355 18 12 7 5 6 3 4
Testing cohort	The Second Affiliated Hospital of Chongqing Medical University	37	66(62-74)	31/6	Pure urothelial carcinoma(PUC) UC with squamous differentiation UC with glandular differentiation	32 1 4
	Chongqing University Fuling Hospital	23	66(55-77)	18/5	Pure urothelial carcinoma(PUC) UC with squamous differentiation UC with glandular differentiation Sarcomatoid UC	14 5 2 2

### Interactive framework and features extraction

**Figure 2:**
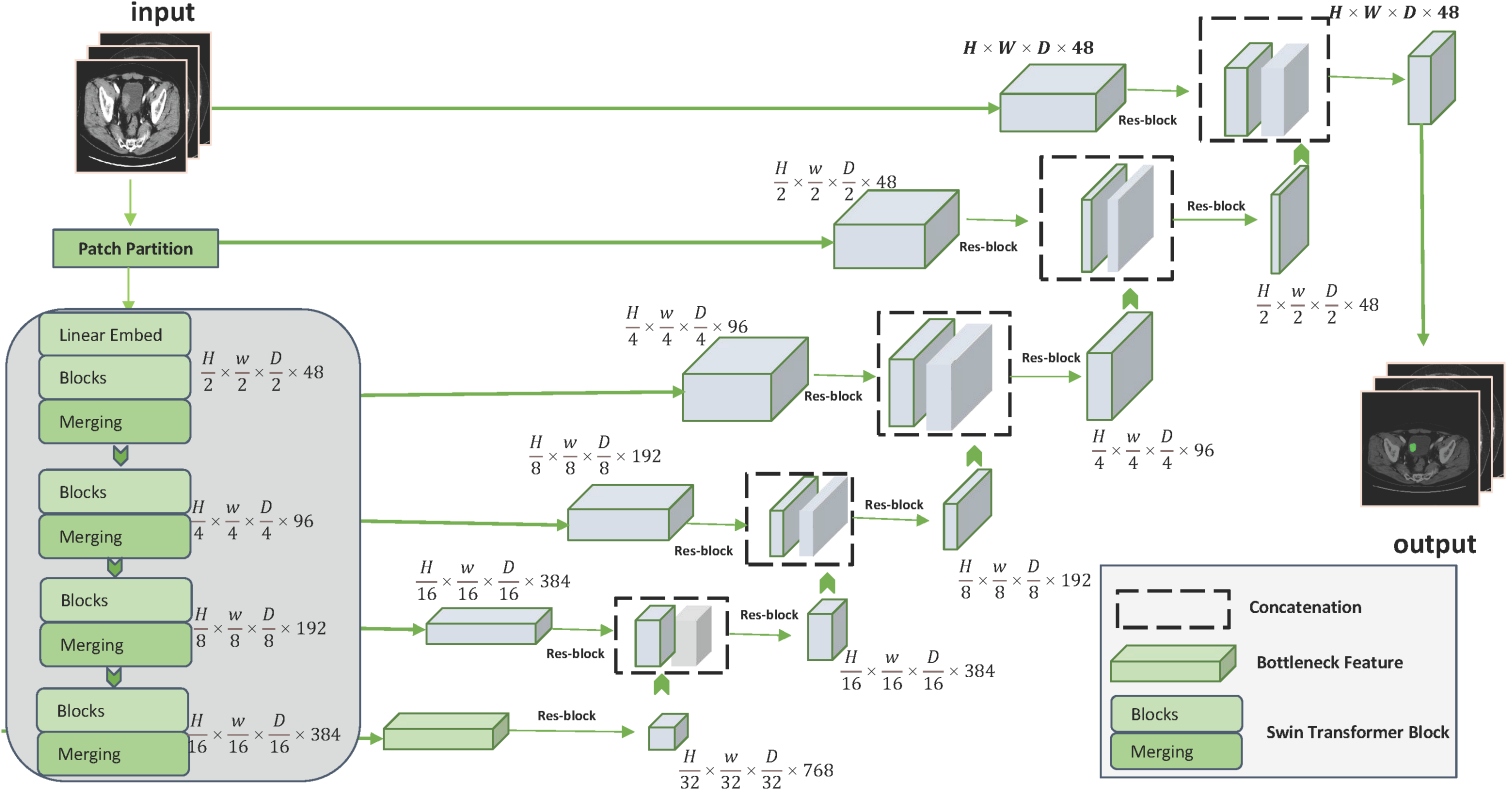
Detailed structure of the Swin UNETR model.

Distinguishing from the traditional manual sketching of regions of interest (ROI) markers to extract features, we constructed an interactive deep learning-based bladder cancer image segmentation framework. The segmentation framework is built on the Swin UNETR algorithm, a novel 3D transformer-based architecture specifically designed for medical image segmentation. Additional Swin UNETR description can be found in **Supplementary Materials**. In our proposed framework, the Swin UNETR model is used to process raw image information and refine the results through user interaction. These user’ interactions are translated into geodesic distance maps, which are then incorporated into the Swin UNETR’s input (26). **Figure 2** provides a comprehensive illustration of the Swin UNETR model’s structure. For this interactive segmentation model’s development, two experienced radiologists (more than 5 years working experience) annotated ROI in bladder cancer images using ITK-SNAP software. They incorporated image data from three different planes—axial, sagittal, and coronal—when delineating tumor margins. In instances of ambiguous sketching, an additional senior radiologist (Lv Fajin, more than 20 years working experience) is consulted to discuss and finalize the results collaboratively. This interactive segmentation model will be used for subsequent extraction of both radiomic features and deep learning features.

Radiomic features are categorized into three types: (1) first-order statistics, (2) shape features, and (3) second-order features. Image types for radiomic features fall into three distinct categories: (1) Original, (2) Log, and (3) Wavelet. Utilizing the default parameters set from the official Pyradiomics YAML file, we successfully extracted 1231 radiomic features for each subject. For the extraction of deep learning features, we employed a 3D-cropbox designed to encapsulate the bladder cancer region. In terms of width and length, the 3D-cropbox correspond to the maximum cross-sectional area of the bladder cancer, while in terms of height, it aligns with the Z-axis dimension that encompass the bladder cancer region. Within the 3D-cropbox, any NumPy array values outside the ROI are set to zero. The 3D-cropbox region is then input into a 3DResnet50 model equipped with weights that have been pretrained. Through removing the pre-trained model’s last layer, disabling gradient updates and adding a 3D maximum pooling layer, we successfully extract 2048 deep learning features per subject. Further details on the 3DResnet50 structure are available in **Supplementary Table S1**. Additionally, **Supplementary Figure S1** displayed the feature screening related processes and **Supplementary Figure S2** provides an in-depth view of the 3D-cropbox workflow. Radiomic features and deep learning features (without clinically relevant data) will be used to build subsequent predictive models.

### Reliability assessment of selected features

To assess the reproducibility and reliability of the selected features, we used the intra-class correlation coefficients and inter-class correlation coefficients (ICC). The inter-class correlation coefficients were calculated based on the re-labeling of 25% participants’ ROIs in both the training and testing cohorts. This re-labeling was performed with the interactive segmentation model by two independent readers. Additionally, these participants were randomly selected by another independent radiologist. As for the intra-class correlation coefficients, they were determined by a single reader who randomly plotted the ROIs of the same participants using the interactive segmentation model in the enrolled datasets twice, with a one-month interval between sessions (27).

### Quality control procedures

The process of quality control for fusion feature extraction and model construction in our study is methodically organized into five distinct sections: (1) quality control of images: ensuring the integrity and appropriateness of images used; (2) quality control of ROI: verifying the accuracy and relevance of the selected ROI; (3) quality control of feature extraction: monitoring the precision and consistency in extracting features; (4) quality control of feature selection: evaluating the criteria and methods used for selecting features; and (5) quality control of machine learning algorithms: reviewing the application and effectiveness of machine learning techniques. In accordance with the Image Biomarker Standardization Initiative (IBSI), we complied with its recommendations (28). To assess the reliability of our research, we adopted the Radiomics quality score (RQS) (29). For more detailed information on our quality control procedures and the results of the RQS calculation, please refer to the **Supplementary Material**.

### Stacking ensemble learning approach

In our study, we employed a stacking ensemble learning approach. Specifically, we used XGBoost, Random Forest (RF), and Decision Tree (DT) as the base models. The predictions from these base models were then used as inputs (meta-features) for the LightGBM model, which served as the meta-learner in our stacking framework. LightGBM was selected for its efficiency and scalability, particularly in handling large datasets and complex feature interactions. By leveraging this hierarchical structure, our stacking ensemble method could capture and combine different levels of information from each base model, leading to more accurate stratification.

### Statistical analysis

**Figure 3:**
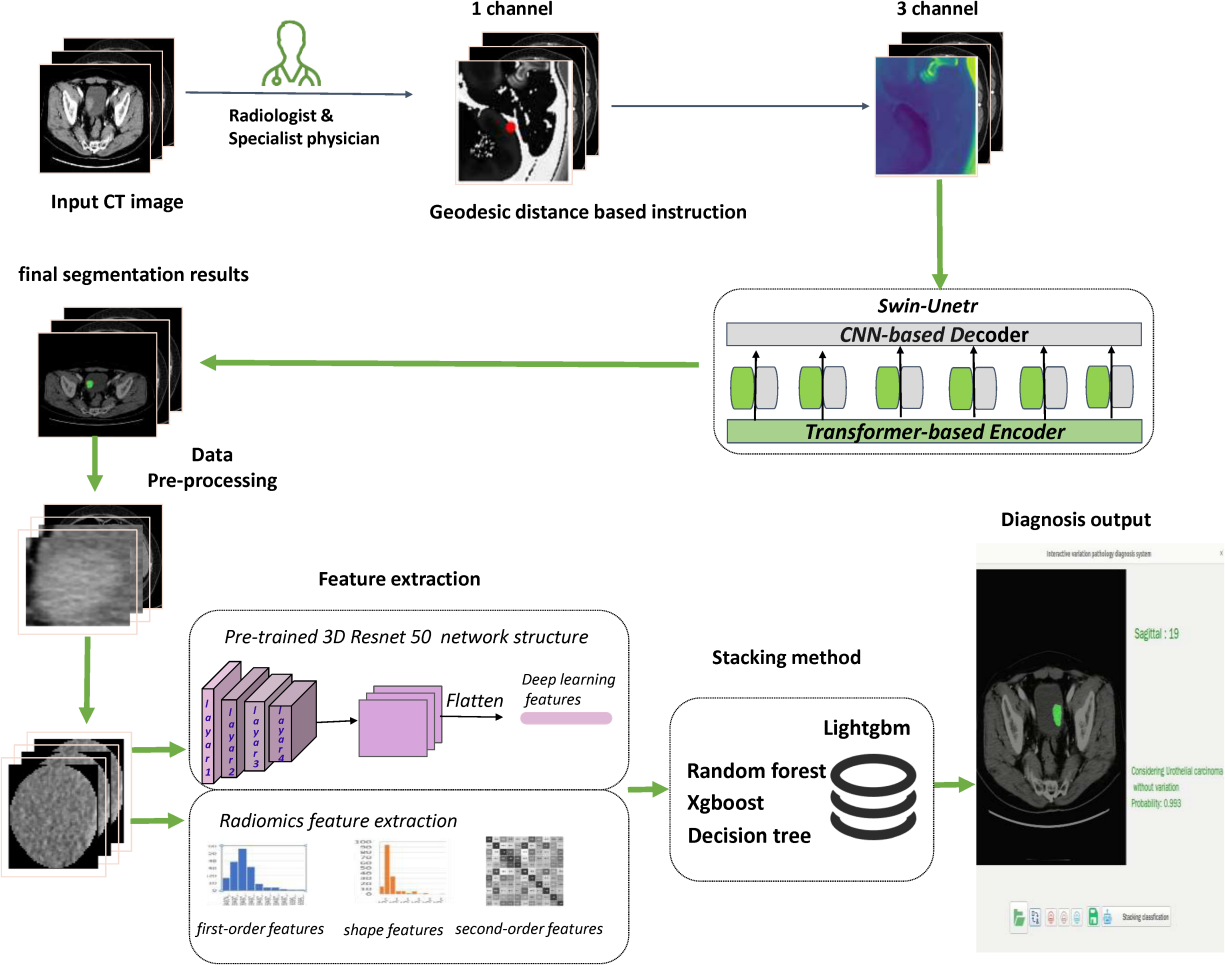
Flowchart presented the step-by-step procedures in machine learning model construction.

We utilized ITK-SNAP (version 3.6.0) to generate ROIs. Pyradiomics package (version 3.0.1) was used to extract radiomic features. The pretrained weights of 3DResnet50 model derived from 23 medical datasets, which included images of various parts of the human body. The pretrained weights file, along with the related codes, are openly available in the Tencent Medicalnet project (https://github.com/Tencent/MedicalNet). The originally chosen radiomics and deep learning features were analyzed for redundancy using Pearson correlation coefficients for normal distributions and Spearman’s rank correlation coefficients for non-normal distributions. **Figure 3** illustrates the entire model construction procedure. To assess the model’s performance, we used the Receiver Operating Characteristics (ROC) curve and the Accuracy Score (ACC). The DeLong test was employed to evaluate if there was significant heterogeneity in the area under the ROC curve (AUC). Additionally, a calibration curve was used to assess the consistency of the final model’s performance in the external validation dataset. Two-sided p values less than 0.05 were considered statistically significant.

The scikit-learn package and “Pycaret” package are used to develop the final machine learning model. All model building processes and plot drawings were conducted in Python (version 3.9) environment and R software (version 4.0.5).

### Ethical approval and consent to participate

We confirm that all methods were carried out in accordance with the relevant guidelines and regulations. This retrospective analysis received approval from the ethics committees of each participating hospital. The written informed consents were waived. All patient information enrolled in this research was anonymized.

## Results

The interactive segmentation model exhibited excellent performance in both training (Dice = 0.78, IOU = 0.65, SEN = 0.83, SPE = 1.00) and testing datasets (Dice = 0.80, IOU = 0.67, SEN = 0.89, SPE = 1.00).

**Figure 4:**
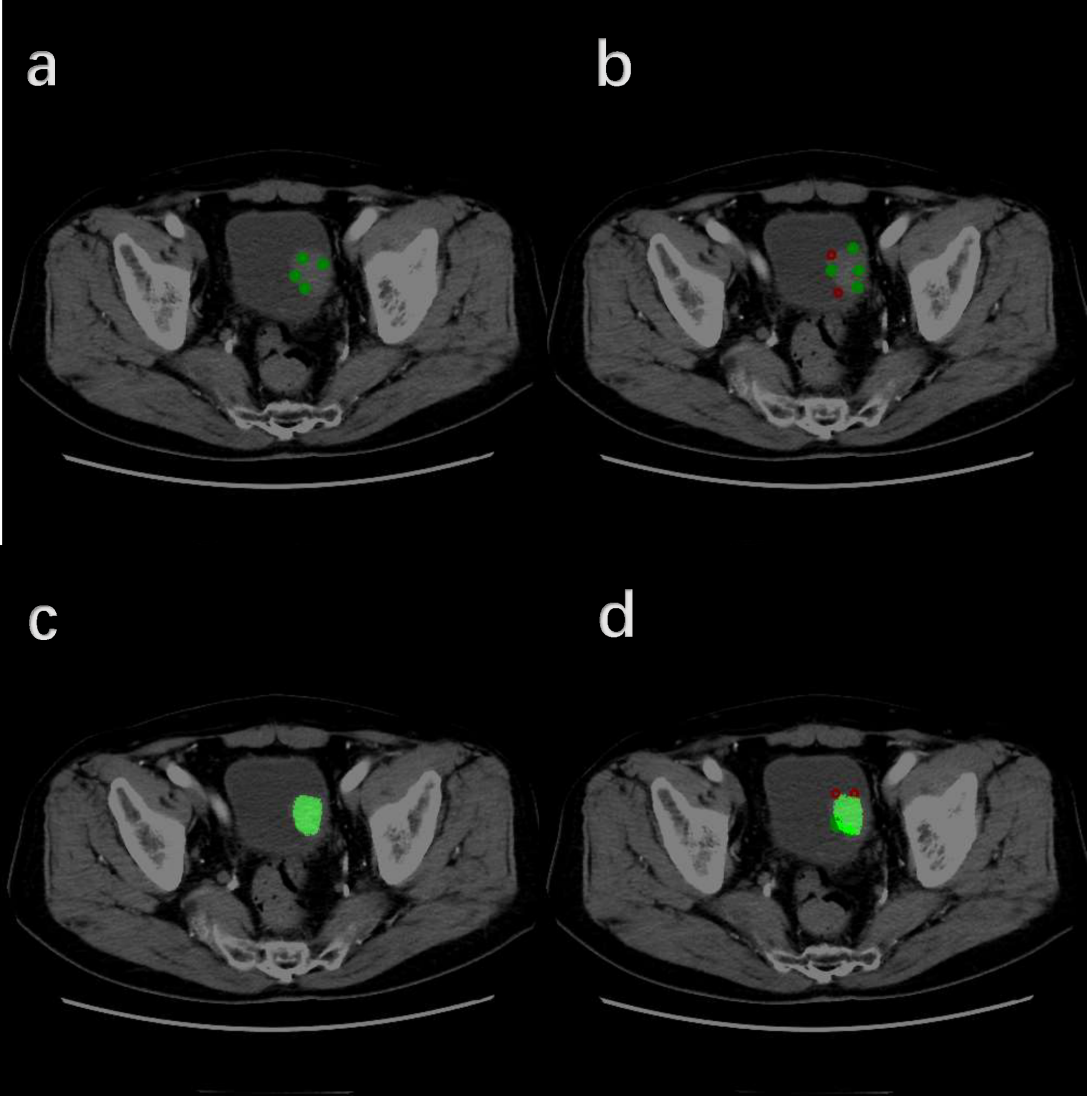
Detailed workflow of the Interactive Segmentation Framework (Operate in order of **A–D**). **(A)** Initial interactive segmentation process combined with positive indicator points (green dots). **(B)** Initial interactive segmentation process combined with negative indicator points (red dots). **(C)** Initial segmentation results. **(D)** Further segmentation refinement.

In the validation set, the Dice of the segmentation model reaches 0.78; in the test set, the Dice of the segmentation model reaches 0.80. These results indicate that our interactive segmentation model exhibits excellent performance in segmentation tasks. **Supplementary Table S2** shows the performance of interactive segmentation model in training and testing datasets in detail. **Figure 4** demonstrates in detail how this segmentation model works.

We initially extracted a total of 3279 features, comprising 1231 radiomic features and 2048 deep learning features. Utilizing the Least Absolute Shrinkage and Selection Operator (LASSO) method for feature screening, we narrowed this down to 64 features. By using Pearson’s correlation coefficient and Spearman’s rank correlation coefficients to check whether there is redundancy in the initial selection of radiomic features and deep learning features, as well as by using intraclass correlation coefficient and interclass correlation coefficient to inspect the reproducibility and reliability of the selected features, 50 features were finally retained, including 46 radiomic features and 4 deep learning features. **Supplementary Table S3** displayed the selected features weights after lasso selection. **Supplementary Table S4** displayed the ICC values in the final selected 46 radiomic features and 4 deep learning features.

**Figure 5:**
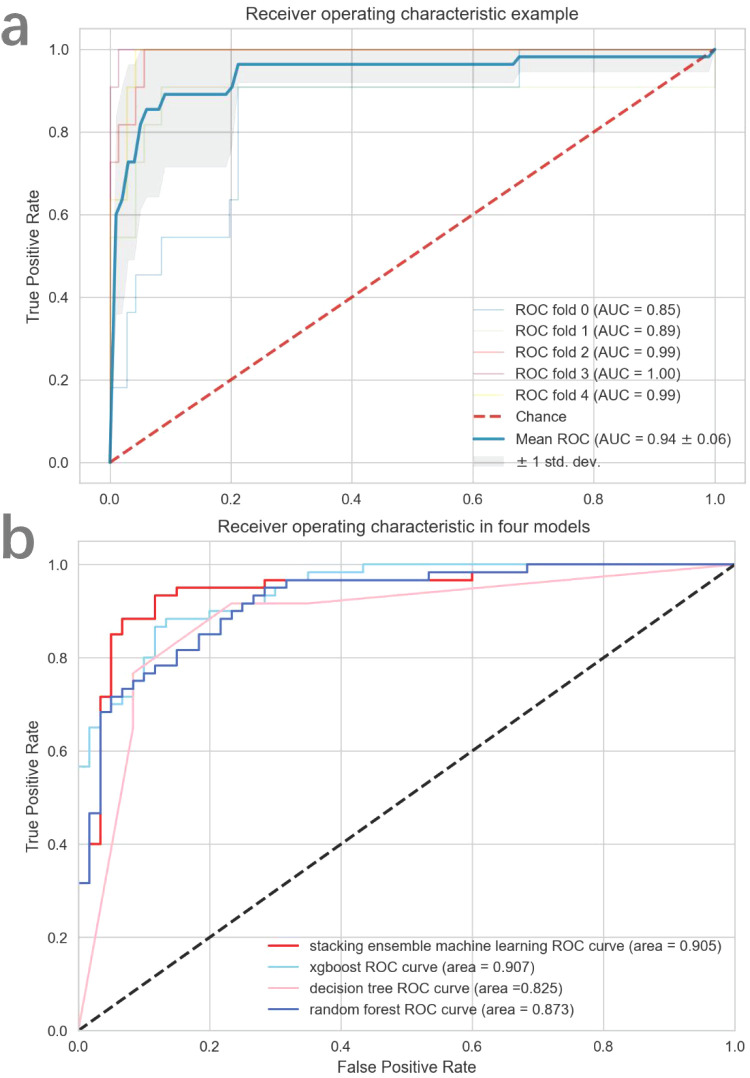
The diagnostic efficacy of each model assessed by ROC curve. In **(A)**, the mean cross-validated ROC of stacking ensemble model was 0.94. In **(B)**, all four models performed excellently in external validation dataset.

**Figure 5** demonstrates a detailed analysis of the performances of the fusion-features based machine learning algorithm. The AUC value for the training cohort is 0.940 and the AUC value for the testing cohort is 0.905. These values indicate that our model not only demonstrates excellent performance in standard evaluations but also maintains robust predictive ability during external validation. Besides, an ACC value of 0.900 was measured in the stacking ensemble model, indicating a high level of discrimination in the diagnosis of urothelial carcinoma with and without histological variants in this study. A comprehensive comparison of the final model’s performance against other common machine learning algorithms is presented in **Table 2**. Moreover, the decision curve analysis applied to external validation datasets reveals that our fusion-feature machine learning model outperforms the “none” and “all” treatment strategies across various threshold probabilities, offering a higher net benefit as shown in **Supplementary Figure S8**. **Supplementary Figures S3, S4** displayed the calibration curve in training and external validation dataset.

**Table:** TABLE 2 The performance of four classifier models.

	Model	AUC (95%CI)	ACC (95%CI)	Sensitivity	Specificity
train cohort 5-fold cross- validation	stacking ensemble	0.940(0.893-0.978)	0.868(0.868-0.869)	0.945(0.885-1.000)	0.856(0.820-0.893)
	xgboost	0.950(0.916-0.983)	0.937(0.936-0.937)	0.836(0.739-0.934)	0.952(0.930-0.974)
	decision tree	0.825(0.758-0.893)	0.910(0.909-0.910)	0.709(0.589-0.829)	0.941(0.916-0.965)
	random forest	0.939(0.901-0.977)	0.910(0.909-0.910)	0.873(0.785-0.961)	0.915(0.887-0.944)
test cohort	stacking ensemble	0.905(0.801-1.000)	0.900(0.897-0.903)	0.857(0.674-1.000)	0.913(0.832-0.994)
	xgboost	0.907(0.822-0.991)	0.883(0.880-0.887)	0.714(0.478-0.951)	0.935(0.863-1.000)
	decision tree	0.825(0.679-0.970)	0.817(0.812-0.822)	0.786(0.571-1.000)	0.826(0.717-0.936)
	random forest	0.873(0.768-0.977)	0.850(0.846-0.854)	0.857(0.674-1.000)	0.848(0.744-0.952)

AUC, Area under the receiver operating characteristic curve; ACC, accuracy score; 95%CI: 95% confidence interval.

## Discussion

In our study, we developed and validated a system that integrates both radiomic and deep learning features extracted from CT images. This system is designed to efficiently and non-invasively identify histological variants of bladder cancer at an early stage. The system incorporates a total of 46 radiometric features and 4 deep learning features, achieving an AUC value of 0.940 for the training cohort, which demonstrates that the model we constructed has excellent classification performance. To mitigate the risk of overfitting, we employed a stacking ensemble machine learning approach coupled with cross-validation methods. This strategy proved effective in maintaining the model’s robustness during the training phase. In addition, we performed external validation of the model and achieved an AUC value of 0.905 and an ACC value of 0.900 in the test cohort, which indicates that our model has strong predictive performance beyond the initial training cohort as well. Not only that, the RQS analysis result of this study yielded a result of 15, which attests to the study’s high quality, trustworthiness, and reproducibility.

To enhance the efficiency of identifying ROIs in bladder tumor-enhanced CT scans, we have employed a deep learning framework designed for interactive segmentation of tumor areas. The results of the interactive framework’s segmentation of bladder tumor regions contain a variety of data such as tumor size, and participate in the construction of subsequent stacking ensemble system. Meanwhile, the segmentation model not only delivers superior segmentation performance, but also achieves a Dice of 0.80 in external validation, which indicates that our constructed segmentation framework has robust generalizability. Obviously, traditional manual segmentation is time-consuming, labor-intensive and often yields unsatisfactory results; Fully automated segmentation frequently falls short in accuracy due to poor image quality and the variability among patients (30). In contrast, our interactive segmentation approach substantially reduces the time required for manual segmentation by radiologists while delivering exceptional results. An efficient interactive segmentation tool is of great importance for practical applications. Not only that, we used a more powerful deep learning algorithm, Swin UNETR, in building this segmentation model, which makes it possible to achieve strong segmentation results with less user interaction and less user time.

Bladder cancer with histological variants tends to be more aggressive and responds less effectively to intravesical and cisplatin-based systemic therapies (11, 31, 32). In non-muscle-invasive bladder cancer (NMIBC), the standard treatment typically involves transurethral resection of the bladder tumor (TURBT) with the subsequent adjuvant intravesical instillation of Bacillus Calmette-Guérin (BCG) or chemotherapeutic agents, as needed (33). However, certain bladder cancer with histological variants has a lower response rate to intravesical bladder therapy compared to pure urothelial carcinoma (32, 34, 35). Adhering to standard treatments in such cases may lead to a poorer prognosis. Therefore, for these patients, early radical cystectomy might be a more effective treatment option (31, 36, 37). In muscle-invasive bladder cancer (MIBC), cisplatin-based combination neoadjuvant chemotherapy followed by radical cystectomy is its main treatment modality (8). However, for certain histological variants of bladder cancer, cisplatin-based combination neoadjuvant chemotherapy is less effective, potentially leading to disease progression if used as the initial treatment (11, 34, 38). In these situations, incorporating immune checkpoint inhibitors or other targeted agents into the neoadjuvant therapy, or opting for direct radical cystectomy, might be more beneficial therapeutic approaches (39, 40). Consequently, the early and accurate identification of histological variants in bladder cancer is crucial for urologist’s clinical decision-making, which significantly impact the prognosis of patients with bladder cancer.

The gold standard for identifying histological variants in bladder cancer relies on the pathologist’s judgment of the pathology specimen (12, 13). However, most of these specimens are obtained through transurethral cystoscopic biopsy or TURBT. This leads to the possibility that the pathology specimens obtained are not all bladder tumor, which in turn affects the pathologist’s ability to accurately identify the presence or absence of histological variants. Furthermore, considering that bladder cancer with histological variants tends to be more aggressive, and the long process of conducting the preoperative examination and waiting for pathological results may lead to tumor progression, which in turn affects patient’s prognosis. In addition, transurethral cystoscopic biopsy or TURBT is an invasive procedure, which in itself is also a harm to the patient. Clearly, the conventional method of determining histological variants of bladder cancer by means of routine pathological biopsy is flawed. There is a pressing need for a simpler, more efficient, and noninvasive method to identify these histological variants of bladder cancer. And the classification model we constructed in this study is just enough to fulfill the existing needs. Moreover, because the AI algorithm can identify a lot of information about the tumor area on CT images, the percentage of histological variants in bladder cancer does not affect the accuracy of the model.

Currently, in the realm of bladder cancer research, there have been a large number of studies evaluating the pathological grading, muscle invasiveness, efficacy and prognosis of bladder cancer through models constructed by machine learning algorithms (22–25, 41, 42), but fewer studies have been conducted in the field of histological variants. Jingwen Huang et al. (43) constructed a machine-learning model based on radiomic features of MRI to predict whether urothelial carcinoma is accompanied by squamous differentiation. Despite the model ultimately achieved excellent predictive performance, only being able to differentiate between urothelial carcinoma with or without squamous differentiation was the primary limitation of the study, as the histological variants of bladder cancer are diverse. For example, when the model is confronted with an MRI image of a urothelial carcinoma with sarcomatoid differentiation, it is bound to give the wrong answer. Moreover, the study only included a sample of 119 cases from a single institution, so the effect of external validation of the model’s performance is uncertain. Sehnaz Evrimler et al. (44) developed a machine learning model using Computed Tomography (CT) to predict histological variants in bladder cancer. However, only a sample of 37 cases of radical cystectomy was included in this study. The authors indicated that the small sample size was attributed to the exclusive focus on patients who underwent radical cystectomy and the absence of preoperative CT-enhanced abdominal scans in some patients. Obviously, our study bridges the gaps existing in previous studies as much as possible. We focus not only on maintaining model performance but also on expanding the sample size and diversifying the sources of our samples, thus enhancing the robustness and applicability of our findings in this critical area of bladder cancer research.

Despite our constructed model demonstrates strong diagnostic performance, the following restrictions exist throughout the study. Firstly, while this study achieved external validation as well as secured an adequate sample size, it’s evident that larger sample sizes could further enhance the construction and generalizability of machine learning models. Secondly, our study solely relied on imaging data for model construction. Therefore, additional research is necessary to determine if integrating clinical and genetic data into the model could enhance its diagnostic performance. Thirdly, our study incorporated data from multiple institutions, where variations in Computed Tomography specifications across these organizations may have influenced the model’s construction. Fourthly, due to the sample size of various types of histological variants, our study was temporarily unable to identify a specific type of histological variants. This is also a key direction of our follow-up research.

## Conclusion

In summary, we constructed a stacking ensemble system for early, non-invasive identification of histological variants in bladder cancer and its model performance achieved excellent results in an external validation set, which will help urologists make clinical decisions.

## Data Availability

The data that support the findings of this study are available on request from the corresponding author upon reasonable request.
