# Supplementary material for: A stacking ensemble system for identifying the presence of histological variants in bladder carcinoma: a multicenter study
Source: Front Oncol. 2025 Jan 10;14:1469427. doi: 10.3389/fonc.2024.1469427 (PMC11757263; doi:10.3389/fonc.2024.1469427)

Supplementary material

**Title:** A stacking ensemble system for identifying the presence of histological variants in bladder carcinoma: a multicenter study

**Author information:**

Canjie Peng^1#^, Quanhao He^1#^, Fajin Lv^2^, Qing Jiang^3^, Yong Chen^4^, Zongjie Wei^1^, Yingjie Xv^1^, Fangtong Liao^1^, Mingzhao Xiao^1*^

^1^ Department of Urology, The First Affiliated Hospital of Chongqing Medical University, Chongqing, China

^2^ Department of Radiology, The First Affiliated Hospital of Chongqing Medical University, Chongqing, China

^3^ Department of Urology, The Second Affiliated Hospital of Chongqing Medical University, Chongqing, China

^4^ Department of Urology, Chongqing University Fuling Hospital, Chongqing, China

# These authors contributed equally to this work.

* Correspondence:

Mingzhao Xiao (Email: mingzhaoxiao@cqmu.edu.cn)

Department of Urology, The First Affiliated Hospital of Chongqing Medical University, Chongqing, China, No. 1 Youyi Road, Chongqing, 400016 Yuzhong, China.

**Content:**

Detailed quality control methods

Additional Swin UNETR description

The detailed 3D-cropbox workflow

The detailed MedicalNet section

Supplementary table S1 Detailed structure components and parameters in 3Dresnet50 model

Supplementary table S2 Performance of interactive segmentation model in training and testing datasets

Supplementary table S3 Selected features weights after lasso selection

Supplementary table S4 The ICC values in the final selected 46 radiomic features and 4 deep learning features

Supplementary figure S1 Feature Screening Related Processes

Supplementary figure S2 The detailed 3D-cropbox workflow

Supplementary figure S3 Calibration curve in the training cohort

Supplementary figure S4 Calibration curve in the external validation cohort

Supplementary figure S5 An example of histopathological image with corresponding CT images

Supplementary figure S6 The confusion matrix for cross-validation set

Supplementary figure S7 The confusion matrix for external validation set

Supplementary figure S8 Decision curve analysis for four machine learning systems in external validation dataset

Supplementary figure S9 heatmap of the correlation coefficients

Supplementary video Interactive segmentation process demonstration (please see supplementary video for details)

Detailed quality control methods

**CT scans quality control**

In training cohort, contrast-enhanced CT scans were obtained from 128-slice spiral CT scanners (Siemens Healthcare, Germany) or 64-slice spiral CT scanners (General Electric, USA). In external validation cohort, enrolled patients underwent contrast-enhanced CT scans with a 128-slice scanner (LightSpeed VCT, GE Medical systems, USA) or 64-slice spiral CT scanners (General Electric, USA). CT data were generated from the standardized scanning protocols. Details are as follows:CT-tube voltage (120-140 kv), CT-tube current (125-300 mAs), scanning matrix (512*512 pixels), body reconstruction kernel and slice thickness (ranging from 1mm to 5mm). After intravenous administration of iohexol (300 mg/mL at a rate of 3.0 mL/s, followed by a 30-mL saline flush), contrast-enhanced CT samples were captured. We retrieved CT images from the corresponding picture archiving and communication systems (Vue PACS, Carestream Health Inc & General Electric Advantage Workstation). The total contrast volume for each kilogram was 1.5 ml. The arterial phases images were selected for further research.

ROI sketching quality control

Considering that manual ROI delineation is vulnerable to wide variation between observers, which may lead to unreliable results, we adhere to the ISBI recommendations for validating the repeatability of ROI regions. Feature reproducibility is evaluated by intra-class correlation coefficients and inter-class correlation coefficients (ICC), which are generated from radiomic features extracted by two independent ROI sketchers. In this study, ICC threshold was set to 0.9 and all the features in the training datasets were normalized prior to feature selection according to their mean value and standard deviation value (z-score normalization) to make sure the comparability of each selected variable (Supplementary table3).

Feature extraction quality control

**preprocessing for 3Dresnet 50 features extraction**

Within the cropped area, the area outside the ROI will be fulfilled with black. After segmentation of the tumor region delineated, the informative slices (the consecutive axial slices containing full tumor area) are cropped and resized to 14 *28 * 28 dimensional NumPy format files (the size for the input layer of the 3Dresnet models). The cropped images will be selected as the input of convolutional neural network (CNN) model.

**3Dresnet 50 network structure**

Tencent Medicalnet's 3Dresnet model, which was pretrained on 23 medical datasets, is employed in this study to extract deep learning features. This model is publicly assessable as open-source code (<https://github.com/Tencent/MedicalNet> ). To extract deep learning features, we modified the 3DResnet50 model by adding a 3D max-pooling layer and removing the upsampling layer. After data pre-processing for deep learning features and model modification, the cropped CT scans were propagated in the network to generate deep learning features.

**Radiomic features parameters settings**

Using the python Pyradiomics package, radiomic features were created by the ROI of our interactive deep learning-based framework. Detailed calculation methods of radiomic features are described and provided in online documentation of Pyradiomics (<https://pyradiomics.readthedocs.io/en/latest/features.html> ). We start features extraction by using the standard sample parameters setting provided in the official Pyradiomics YAML file and all the images will be resampled to 1×1×1 mm³ voxels to standardize the slice thickness.(1). Image intensities will be binned by 25 HU and voxel array shift is set to 1000. All radiomic features included in this investigation adhere to the Imaging Biomarker Standardization Initiative's feature criteria (IBSI).

Candidate features selection quality control

Radiomic features extraction was conducted using Pyradiomics package (version 3.0.1) in python environment (version 3.7). 1231 radiomic features and 2048 deep learning features were generated in each individual at the beginning. After feature extraction, the least absolute shrinkage and selection operator (LASSO) method with 10-fold cross-validation was adapted to identify the most significant features in the training datasets. All candidate variables were normalized before LASSO selection. At the selected λ value of 0.0154, 64 candidate features were selected by LASSO methods. To eliminate redundancy in the primary selected candidate variables, the Spearman's correlation coefficient for variables with non-normal distribution and the Pearson correlation coefficient for variables with normal distribution were separately employed.

Machine learning algorithms quality control

Detailed radiomics quality score (RQS) calculation results

The radiomics quality score (RQS1.0 version) of this study reached 15(2). The cumulated points are obtained by complying with image protocol quality (+1), feature reduction or adjustment for multiple testing (+3), discrimination method with resampling method (+1), calibration statistics method (+1), validation from another institute (+4), comparison to “gold standard” (+2), potential clinical utility (+2), open-sourced code (+1).

Correctness of each model and the corresponding confusion matrix

All four models showed well performance and satisfactory accuracy score (decision tree ACC=81.7%, random forest ACC=85.0%, xgboost ACC=88.3%, stacking ensemble algorithm ACC =90.0%).

Reference:

1.van Griethuysen, J. J. M., Fedorov, A., Parmar, C., Hosny, A., Aucoin, N., Narayan, V., Beets-Tan, R. G. H., Fillion-Robin, J.-C., Pieper, S., and Aerts, H. J. W. L. (2017) Computational Radiomics System to Decode the Radiographic Phenotype. *Cancer Research* **77**, e104-e107

2.Lambin, P., Leijenaar, R. T. H., Deist, T. M., Peerlings, J., de Jong, E. E. C., van Timmeren, J., Sanduleanu, S., Larue, R. T. H. M., Even, A. J. G., Jochems, A., van Wijk, Y., Woodruff, H., van Soest, J., Lustberg, T., Roelofs, E., van Elmpt, W., Dekker, A., Mottaghy, F. M., Wildberger, J. E., and Walsh, S. (2017) Radiomics: the bridge between medical imaging and personalized medicine. Nature Reviews Clinical Oncology 14, 749-762

**Additional Swin UNETR description**

This architecture comprises a Swin Transformer encoder and a U-Net decoder. The Swin Transformer encoder is akin to the Swin Transformer employed in natural language processing tasks, yet it has been modified to adapt for 3D data. As the encoder begins, it creates non-overlapping patches from the input data. Utilizing a patch partition layer, these are further processed to create predetermined-sized windows, facilitating the computation of self-attention. This encoder then contains six Swin Transformer blocks, each utilizing a different window size. These blocks employ a hierarchical attention mechanism to capture long-range dependencies within the data. Correspondingly, the U-Net decoder follows the standard U-Net architecture, tailored to assimilate the output from the Swin Transformer encoder. It starts with a 1x1x1 convolution to upsample the encoder's output back to the original input size and includes six U-Net blocks, each with varying output channels. These blocks leverage skip connections from the encoder to the decoder, aiding in the preservation of spatial information.

The detailed 3D-cropbox workflow

In the detailed 3D-cropbox workflow, there are three main components: cropping the ROI areas, filling the background regions, and saving the cropped images.  The areas outside the ROI are filled with black.  When extracting deep learning features, all ROI areas are resized to a fixed size according to MedicalNet’s input rules (input_D = 14, input_H = 28, input_W = 28).  For radiomic feature extraction, the features are derived from the original and segmented images.  This process follows the pyradiomics official CT feature extraction YAML file(https://github.com/AIM-Harvard/pyradiomics/blob/master/examples/exampleSettings/exampleCT.yaml), using the radiomics package for feature extraction.  Therefore, differences in volume sizes and shapes do not affect the radiomic or deep learning feature extraction process.  In fact, the differences in volume sizes and shapes, as important classification features, are preserved and used in the subsequent classification modeling.

The detailed MedicalNet section

Unlike traditional 2D-resnet 50 using a single RGB image, the 3D-resnet 50 model (Tencent Medicalnet) add an additional channel which can hold whole spatial information in ROI area. The reason why we select Tencent Medicalnet model for transfer-learning is that it was pretrained on 23 medical datasets (including brain MR images and lung CT images, etc.), Unlike previous widely used transfer-learning CNN model which were pre-trained on the non-medical database like ImageNet. The 3D-resnet 50 model and corresponding pre-training weights are open-source and freely available in github (https://github.com/Tencent/MedicalNet). The reason for selecting MedicalNet for deep learning features extraction instead of other self-supervised pretrained models is that many of them are based on 2D images, whereas the 3D-resnet based Medicalnet model demonstrated excellent performance in several medical challenge datasets. The detailed network performance has been thoroughly described in the article "Med3D: Transfer Learning for 3D Medical Image Analysis" (DOI: https://doi.org/10.48550/arXiv.1904.00625).

**Supplementary table S1** Detailed structure components and parameters in 3Dresnet50 model

| **3DResnet model structure** | | | | | |
| --- | --- | --- | --- | --- | --- |
| **Layer (type)** | **Output shape** | **Parameters** | **Layer (type)** | **Output Shape** | **Parameters** |
| Conv3d-1 | [-1, 64, 7, 64, 64] | 21,952 | BatchNorm3d-88 | [-1, 1024, 2, 16, 16] | 2,048 |
| BatchNorm3d-2 | [-1, 64, 7, 64, 64] | 128 | ReLU-89 | [-1, 1024, 2, 16, 16] | 0 |
| ReLU-3 | [-1, 64, 7, 64, 64] | 0 | Bottleneck-90 | [-1, 1024, 2, 16, 16] | 0 |
| MaxPool3d-4 | [-1, 64, 4, 32, 32] | 0 | Conv3d-91 | [-1, 256, 2, 16, 16] | 262,144 |
| Conv3d-5 | [-1, 64, 4, 32, 32] | 4,096 | BatchNorm3d-92 | [-1, 256, 2, 16, 16] | 512 |
| BatchNorm3d-6 | [-1, 64, 4, 32, 32] | 128 | ReLU-93 | [-1, 256, 2, 16, 16] | 0 |
| ReLU-7 | [-1, 64, 4, 32, 32] | 0 | Conv3d-94 | [-1, 256, 2, 16, 16] | 1,769,472 |
| Conv3d-8 | [-1, 64, 4, 32, 32] | 110,592 | BatchNorm3d-95 | [-1, 256, 2, 16, 16] | 512 |
| BatchNorm3d-9 | [-1, 64, 4, 32, 32] | 128 | ReLU-96 | [-1, 256, 2, 16, 16] | 0 |
| ReLU-10 | [-1, 64, 4, 32, 32] | 0 | Conv3d-97 | [-1, 1024, 2, 16, 16] | 262,144 |
| Conv3d-11 | [-1, 256, 4, 32, 32] | 16,384 | BatchNorm3d-98 | [-1, 1024, 2, 16, 16] | 2,048 |
| BatchNorm3d-12 | [-1, 256, 4, 32, 32] | 512 | ReLU-99 | [-1, 1024, 2, 16, 16] | 0 |
| Conv3d-13 | [-1, 256, 4, 32, 32] | 16,384 | Bottleneck-100 | [-1, 1024, 2, 16, 16] | 0 |
| BatchNorm3d-14 | [-1, 256, 4, 32, 32] | 512 | Conv3d-101 | [-1, 256, 2, 16, 16] | 262,144 |
| ReLU-15 | [-1, 256, 4, 32, 32] | 0 | BatchNorm3d-102 | [-1, 256, 2, 16, 16] | 512 |
| Bottleneck-16 | [-1, 256, 4, 32, 32] | 0 | ReLU-103 | [-1, 256, 2, 16, 16] | 0 |
| Conv3d-17 | [-1, 64, 4, 32, 32] | 16,384 | Conv3d-104 | [-1, 256, 2, 16, 16] | 1,769,472 |
| BatchNorm3d-18 | [-1, 64, 4, 32, 32] | 128 | BatchNorm3d-105 | [-1, 256, 2, 16, 16] | 512 |
| ReLU-19 | [-1, 64, 4, 32, 32] | 0 | ReLU-106 | [-1, 256, 2, 16, 16] | 0 |
| Conv3d-20 | [-1, 64, 4, 32, 32] | 110,592 | Conv3d-107 | [-1, 1024, 2, 16, 16] | 262,144 |
| BatchNorm3d-21 | [-1, 64, 4, 32, 32] | 128 | BatchNorm3d-108 | [-1, 1024, 2, 16, 16] | 2,048 |
| ReLU-22 | [-1, 64, 4, 32, 32] | 0 | ReLU-109 | [-1, 1024, 2, 16, 16] | 0 |
| Conv3d-23 | [-1, 256, 4, 32, 32] | 16,384 | Bottleneck-110 | [-1, 1024, 2, 16, 16] | 0 |
| BatchNorm3d-24 | [-1, 256, 4, 32, 32] | 512 | Conv3d-111 | [-1, 256, 2, 16, 16] | 262,144 |
| ReLU-25 | [-1, 256, 4, 32, 32] | 0 | BatchNorm3d-112 | [-1, 256, 2, 16, 16] | 512 |
| Bottleneck-26 | [-1, 256, 4, 32, 32] | 0 | ReLU-113 | [-1, 256, 2, 16, 16] | 0 |
| Conv3d-27 | [-1, 64, 4, 32, 32] | 16,384 | Conv3d-114 | [-1, 256, 2, 16, 16] | 1,769,472 |
| BatchNorm3d-28 | [-1, 64, 4, 32, 32] | 128 | BatchNorm3d-115 | [-1, 256, 2, 16, 16] | 512 |
| ReLU-29 | [-1, 64, 4, 32, 32] | 0 | ReLU-116 | [-1, 256, 2, 16, 16] | 0 |
| Conv3d-30 | [-1, 64, 4, 32, 32] | 110,592 | Conv3d-117 | [-1, 1024, 2, 16, 16] | 262,144 |
| BatchNorm3d-31 | [-1, 64, 4, 32, 32] | 128 | BatchNorm3d-118 | [-1, 1024, 2, 16, 16] | 2,048 |
| ReLU-32 | [-1, 64, 4, 32, 32] | 0 | ReLU-119 | [-1, 1024, 2, 16, 16] | 0 |
| Conv3d-33 | [-1, 256, 4, 32, 32] | 16,384 | Bottleneck-120 | [-1, 1024, 2, 16, 16] | 0 |
| BatchNorm3d-34 | [-1, 256, 4, 32, 32] | 512 | Conv3d-121 | [-1, 256, 2, 16, 16] | 262,144 |
| ReLU-35 | [-1, 256, 4, 32, 32] | 0 | BatchNorm3d-122 | [-1, 256, 2, 16, 16] | 512 |
| Bottleneck-36 | [-1, 256, 4, 32, 32] | 0 | ReLU-123 | [-1, 256, 2, 16, 16] | 0 |
| Conv3d-37 | [-1, 128, 4, 32, 32] | 32,768 | Conv3d-124 | [-1, 256, 2, 16, 16] | 1,769,472 |
| BatchNorm3d-38 | [-1, 128, 4, 32, 32] | 256 | BatchNorm3d-125 | [-1, 256, 2, 16, 16] | 512 |
| ReLU-39 | [-1, 128, 4, 32, 32] | 0 | ReLU-126 | [-1, 256, 2, 16, 16] | 0 |
| Conv3d-40 | [-1, 128, 2, 16, 16] | 442,368 | Conv3d-127 | [-1, 1024, 2, 16, 16] | 262,144 |
| BatchNorm3d-41 | [-1, 128, 2, 16, 16] | 256 | BatchNorm3d-128 | [-1, 1024, 2, 16, 16] | 2,048 |
| ReLU-42 | [-1, 128, 2, 16, 16] | 0 | ReLU-129 | [-1, 1024, 2, 16, 16] | 0 |
| Conv3d-43 | [-1, 512, 2, 16, 16] | 65,536 | Bottleneck-130 | [-1, 1024, 2, 16, 16] | 0 |
| BatchNorm3d-44 | [-1, 512, 2, 16, 16] | 1,024 | Conv3d-131 | [-1, 256, 2, 16, 16] | 262,144 |
| Conv3d-45 | [-1, 512, 2, 16, 16] | 131,072 | BatchNorm3d-132 | [-1, 256, 2, 16, 16] | 512 |
| BatchNorm3d-46 | [-1, 512, 2, 16, 16] | 1,024 | ReLU-133 | [-1, 256, 2, 16, 16] | 0 |
| ReLU-47 | [-1, 512, 2, 16, 16] | 0 | Conv3d-134 | [-1, 256, 2, 16, 16] | 1,769,472 |
| Bottleneck-48 | [-1, 512, 2, 16, 16] | 0 | BatchNorm3d-135 | [-1, 256, 2, 16, 16] | 512 |
| Conv3d-49 | [-1, 128, 2, 16, 16] | 65,536 | ReLU-136 | [-1, 256, 2, 16, 16] | 0 |
| BatchNorm3d-50 | [-1, 128, 2, 16, 16] | 256 | Conv3d-137 | [-1, 1024, 2, 16, 16] | 262,144 |
| ReLU-51 | [-1, 128, 2, 16, 16] | 0 | BatchNorm3d-138 | [-1, 1024, 2, 16, 16] | 2,048 |
| Conv3d-52 | [-1, 128, 2, 16, 16] | 442,368 | ReLU-139 | [-1, 1024, 2, 16, 16] | 0 |
| BatchNorm3d-53 | [-1, 128, 2, 16, 16] | 256 | Bottleneck-140 | [-1, 1024, 2, 16, 16] | 0 |
| ReLU-54 | [-1, 128, 2, 16, 16] | 0 | Conv3d-141 | [-1, 512, 2, 16, 16] | 524,288 |
| Conv3d-55 | [-1, 512, 2, 16, 16] | 65,536 | BatchNorm3d-142 | [-1, 512, 2, 16, 16] | 1,024 |
| BatchNorm3d-56 | [-1, 512, 2, 16, 16] | 1,024 | ReLU-143 | [-1, 512, 2, 16, 16] | 0 |
| ReLU-57 | [-1, 512, 2, 16, 16] | 0 | Conv3d-144 | [-1, 512, 2, 16, 16] | 7,077,888 |
| Bottleneck-58 | [-1, 512, 2, 16, 16] | 0 | BatchNorm3d-145 | [-1, 512, 2, 16, 16] | 1,024 |
| Conv3d-59 | [-1, 128, 2, 16, 16] | 65,536 | ReLU-146 | [-1, 512, 2, 16, 16] | 0 |
| BatchNorm3d-60 | [-1, 128, 2, 16, 16] | 256 | Conv3d-147 | [-1, 2048, 2, 16, 16] | 1,048,576 |
| ReLU-61 | [-1, 128, 2, 16, 16] | 0 | BatchNorm3d-148 | [-1, 2048, 2, 16, 16] | 4,096 |
| Conv3d-62 | [-1, 128, 2, 16, 16] | 442,368 | Conv3d-149 | [-1, 2048, 2, 16, 16] | 2,097,152 |
| BatchNorm3d-63 | [-1, 128, 2, 16, 16] | 256 | BatchNorm3d-150 | [-1, 2048, 2, 16, 16] | 4,096 |
| ReLU-64 | [-1, 128, 2, 16, 16] | 0 | ReLU-151 | [-1, 2048, 2, 16, 16] | 0 |
| Conv3d-65 | [-1, 512, 2, 16, 16] | 65,536 | Bottleneck-152 | [-1, 2048, 2, 16, 16] | 0 |
| BatchNorm3d-66 | [-1, 512, 2, 16, 16] | 1,024 | Conv3d-153 | [-1, 512, 2, 16, 16] | 1,048,576 |
| ReLU-67 | [-1, 512, 2, 16, 16] | 0 | BatchNorm3d-154 | [-1, 512, 2, 16, 16] | 1,024 |
| Bottleneck-68 | [-1, 512, 2, 16, 16] | 0 | ReLU-155 | [-1, 512, 2, 16, 16] | 0 |
| Conv3d-69 | [-1, 128, 2, 16, 16] | 65,536 | Conv3d-156 | [-1, 512, 2, 16, 16] | 7,077,888 |
| BatchNorm3d-70 | [-1, 128, 2, 16, 16] | 256 | BatchNorm3d-157 | [-1, 512, 2, 16, 16] | 1,024 |
| ReLU-71 | [-1, 128, 2, 16, 16] | 0 | ReLU-158 | [-1, 512, 2, 16, 16] | 0 |
| Conv3d-72 | [-1, 128, 2, 16, 16] | 442,368 | Conv3d-159 | [-1, 2048, 2, 16, 16] | 1,048,576 |
| BatchNorm3d-73 | [-1, 128, 2, 16, 16] | 256 | BatchNorm3d-160 | [-1, 2048, 2, 16, 16] | 4,096 |
| ReLU-74 | [-1, 128, 2, 16, 16] | 0 | ReLU-161 | [-1, 2048, 2, 16, 16] | 0 |
| Conv3d-75 | [-1, 512, 2, 16, 16] | 65,536 | Bottleneck-162 | [-1, 2048, 2, 16, 16] | 0 |
| BatchNorm3d-76 | [-1, 512, 2, 16, 16] | 1,024 | Conv3d-163 | [-1, 512, 2, 16, 16] | 1,048,576 |
| ReLU-77 | [-1, 512, 2, 16, 16] | 0 | BatchNorm3d-164 | [-1, 512, 2, 16, 16] | 1,024 |
| Bottleneck-78 | [-1, 512, 2, 16, 16] | 0 | ReLU-165 | [-1, 512, 2, 16, 16] | 0 |
| Conv3d-79 | [-1, 256, 2, 16, 16] | 131,072 | Conv3d-166 | [-1, 512, 2, 16, 16] | 7,077,888 |
| BatchNorm3d-80 | [-1, 256, 2, 16, 16] | 512 | BatchNorm3d-167 | [-1, 512, 2, 16, 16] | 1,024 |
| ReLU-81 | [-1, 256, 2, 16, 16] | 0 | ReLU-168 | [-1, 512, 2, 16, 16] | 0 |
| Conv3d-82 | [-1, 256, 2, 16, 16] | 1,769,472 | Conv3d-169 | [-1, 2048, 2, 16, 16] | 1,048,576 |
| BatchNorm3d-83 | [-1, 256, 2, 16, 16] | 512 | BatchNorm3d-170 | [-1, 2048, 2, 16, 16] | 4,096 |
| ReLU-84 | [-1, 256, 2, 16, 16] | 0 | ReLU-171 | [-1, 2048, 2, 16, 16] | 0 |
| Conv3d-85 | [-1, 1024, 2, 16, 16] | 262,144 | Bottleneck-172 | [-1, 2048, 2, 16, 16] | 0 |
| BatchNorm3d-86 | [-1, 1024, 2, 16, 16] | 2,048 | AdaptiveMaxPool3d-173 | [-1, 2048, 1, 1, 1] | 0 |
| Conv3d-87 | [-1, 1024, 2, 16, 16] | 524,288 | ResNet-174 | [-1, 2048, 1, 1, 1] | 0 |

**Supplementary table S2** Performance of interactive segmentation model in training and testing datasets

| **Cohort** | **Dice (%)** | **IOU (%)** | **SEN (%)** | **SPE (%)** |
| --- | --- | --- | --- | --- |
| training cohort | 77.75±11.63 | 64.91±13.96 | 82.90±9.29 | 99.98±0.02 |
| test cohort | 79.67±9.42 | 67.14±12.27 | 88.55±8.93 | 99.99±0.01 |

**legend: Dice:** dice similarity, SEN: Sensitivity, SPE: specificity. All metrics are presented as mean and standard deviation.

**Supplementary table S3** Selected features weights after lasso selection

| **Feature** | **Weighted Value** |
| --- | --- |
| log.sigma.2.0.mm.3D_glcm_MaximumProbability | -0.123478577 |
| log.sigma.3.0.mm.3D_glrlm_ShortRunEmphasis | -0.086380534 |
| wavelet.LLH_glcm_ClusterShade | -0.064152621 |
| wavelet.LLL_firstorder_10Percentile | -0.062621236 |
| wavelet.LLL_glrlm_ShortRunEmphasis | -0.052313119 |
| log.sigma.2.0.mm.3D_glcm_Imc1 | -0.040851589 |
| log.sigma.4.0.mm.3D_firstorder_Uniformity | -0.035364818 |
| X3dresnet.feature831 | -0.021737803 |
| log.sigma.5.0.mm.3D_glrlm_RunVariance | -0.019105317 |
| wavelet.HLH_glszm_SizeZoneNonUniformity | -0.019103456 |
| wavelet.LLH_glszm_HighGrayLevelZoneEmphasis | -0.018870898 |
| wavelet.LLL_glcm_Imc1 | -0.016362816 |
| wavelet.HHL_glszm_LargeAreaLowGrayLevelEmphasis | -0.015634663 |
| log.sigma.2.0.mm.3D_glcm_InverseVariance | -0.014679934 |
| diagnostics_Mask.original_VolumeNum | -0.014172547 |
| log.sigma.3.0.mm.3D_glcm_MaximumProbability | -0.013613130 |
| log.sigma.4.0.mm.3D_glszm_LargeAreaLowGrayLevelEmphasis | -0.011767885 |
| original_shape_Flatness | -0.011547586 |
| wavelet.LLH_glcm_Imc2 | -0.011545185 |
| wavelet.HHH_glcm_Autocorrelation | -0.011412243 |
| wavelet.LHH_glszm_LowGrayLevelZoneEmphasis | -0.009814121 |
| log.sigma.4.0.mm.3D_glrlm_LongRunLowGrayLevelEmphasis | -0.009772158 |
| log.sigma.5.0.mm.3D_glszm_ZoneEntropy | -0.009427205 |
| wavelet.HHH_glszm_SizeZoneNonUniformity | -0.009212566 |
| wavelet.LHH_firstorder_Mean | -0.008866226 |
| wavelet.LLH_firstorder_RobustMeanAbsoluteDeviation | -0.008502487 |
| wavelet.LLL_gldm_SmallDependenceHighGrayLevelEmphasis | -0.007742268 |
| X3dresnet.feature1029 | -0.007284295 |
| log.sigma.5.0.mm.3D_glszm_SmallAreaLowGrayLevelEmphasis | -0.006993465 |
| log.sigma.2.0.mm.3D_firstorder_90Percentile | -0.005375524 |
| log.sigma.3.0.mm.3D_glszm_GrayLevelNonUniformity | -0.004769000 |
| wavelet.HHH_glszm_ZoneVariance | -0.004412535 |
| wavelet.LHH_glszm_SizeZoneNonUniformity | -0.003592125 |
| X3dresnet.feature1021 | -0.002748047 |
| wavelet.LLH_firstorder_90Percentile | -0.002671890 |
| log.sigma.1.0.mm.3D_glszm_GrayLevelNonUniformityNormalized | -0.002080814 |
| log.sigma.5.0.mm.3D_firstorder_Kurtosis | -0.001776317 |
| wavelet.HHH_glszm_LowGrayLevelZoneEmphasis | -0.000000317 |
| wavelet.LHL_glcm_InverseVariance | 0.000712608 |
| log.sigma.3.0.mm.3D_glszm_SizeZoneNonUniformityNormalized | 0.000814558 |
| wavelet.LHH_glszm_GrayLevelNonUniformityNormalized | 0.001276351 |
| wavelet.HLH_firstorder_Skewness | 0.001507515 |
| wavelet.LLH_glrlm_ShortRunLowGrayLevelEmphasis | 0.001669557 |
| log.sigma.2.0.mm.3D_glszm_SmallAreaLowGrayLevelEmphasis | 0.001779280 |
| wavelet.HLH_glrlm_ShortRunEmphasis | 0.002156658 |
| log.sigma.1.0.mm.3D_glszm_SmallAreaEmphasis | 0.002416461 |
| log.sigma.1.0.mm.3D_glszm_SizeZoneNonUniformityNormalized | 0.002522028 |
| wavelet.HHH_glszm_GrayLevelNonUniformityNormalized | 0.003241749 |
| wavelet.HHL_glszm_SmallAreaEmphasis | 0.003528547 |
| log.sigma.4.0.mm.3D_glszm_SizeZoneNonUniformityNormalized | 0.003651981 |
| wavelet.HHL_glcm_InverseVariance | 0.004240429 |
| wavelet.LLH_glrlm_RunPercentage | 0.005581234 |
| wavelet.HLH_glcm_JointEntropy | 0.006125813 |
| X3dresnet.feature1041 | 0.008920094 |
| log.sigma.1.0.mm.3D_glrlm_GrayLevelNonUniformityNormalized | 0.009668854 |
| wavelet.LHH_firstorder_Minimum | 0.010774586 |
| wavelet.LLH_glszm_SmallAreaLowGrayLevelEmphasis | 0.012212645 |
| wavelet.HHH_glcm_Imc2 | 0.013862226 |
| wavelet.LLH_firstorder_Kurtosis | 0.014186605 |
| wavelet.HLL_gldm_LargeDependenceLowGrayLevelEmphasis | 0.021929670 |
| log.sigma.4.0.mm.3D_glszm_SmallAreaLowGrayLevelEmphasis | 0.022582814 |
| wavelet.LHL_gldm_DependenceVariance | 0.025657469 |
| wavelet.HHH_glszm_HighGrayLevelZoneEmphasis | 0.027858187 |
| wavelet.LLL_firstorder_InterquartileRange | 0.032004070 |

Supplementary table S4 The ICC values in the final selected 46 radiomic features and 4 deep learning features

| **feature label** | **ICC (interclass)** | **ICC (intraclass)** |
| --- | --- | --- |
| log.sigma.2.0.mm.3D_glcm_MaximumProbability | 0.902(0.859-0.932) | 0.902(0.859-0.932) |
| log.sigma.3.0.mm.3D_glrlm_ShortRunEmphasis | 0.954(0.933-0.969) | 0.954(0.933-0.969) |
| wavelet.LLH_glcm_ClusterShade | 0.960(0.942-0.972) | 0.960(0.941-0.973) |
| wavelet.LLL_firstorder_10Percentile | 0.903(0.861-0.932) | 0.901(0.858-0.931) |
| log.sigma.2.0.mm.3D_glcm_Imc1 | 0.783(0.698-0.845) | 0.781(0.695-0.844) |
| log.sigma.4.0.mm.3D_firstorder_Uniformity | 0.961(0.942-0.973) | 0.961(0.942-0.973) |
| X3dresnet.feature831 | 0.976(0.965-0.983) | 0.976(0.966-0.983) |
| log.sigma.5.0.mm.3D_glrlm_RunVariance | 0.999(0.998-1.000) | 0.999(0.998-1.000) |
| wavelet.HLH_glszm_SizeZoneNonUniformity | 0.990(0.985-0.993) | 0.990(0.986-0.993) |
| wavelet.LLH_glszm_HighGrayLevelZoneEmphasis | 0.993(0.989-0.995) | 0.993(0.989-0.995) |
| wavelet.LLL_glcm_Imc1 | 0.880(0.826-0.917) | 0.880(0.826-0.917) |
| wavelet.HHL_glszm_LargeAreaLowGrayLevelEmphasis | 0.998(0.997-0.999) | 0.998(0.997-0.999) |
| log.sigma.2.0.mm.3D_glcm_InverseVariance | 0.991(0.987-0.994) | 0.992(0.989-0.995) |
| original_shape_Flatness | 0.996(0.994-0.997) | 0.996(0.994-0.997) |
| wavelet.LLH_glcm_Imc2 | 0.956(0.938-0.969) | 0.955(0.936-0.969) |
| wavelet.HHH_glcm_Autocorrelation | 0.988(0.982-0.991) | 0.989(0.984-0.992) |
| wavelet.LHH_glszm_LowGrayLevelZoneEmphasis | 0.883(0.836-0.917) | 0.893(0.850-0.925) |
| log.sigma.4.0.mm.3D_glrlm_LongRunLowGrayLevelEmphasis | 0.998(0.997-0.999) | 0.998(0.997-0.999) |
| log.sigma.5.0.mm.3D_glszm_ZoneEntropy | 0.997(0.996-0.998) | 0.997(0.996-0.998) |
| wavelet.LHH_firstorder_Mean | 0.997(0.996-0.998) | 0.997(0.996-0.998) |
| X3dresnet.feature1029 | 0.986(0.981-0.991) | 0.986(0.981-0.991) |
| log.sigma.5.0.mm.3D_glszm_SmallAreaLowGrayLevelEmphasis | 0.988(0.983-0.992) | 0.990(0.985-0.993) |
| log.sigma.3.0.mm.3D_glszm_GrayLevelNonUniformity | 0.989(0.985-0.993) | 0.990(0.986-0.993) |
| wavelet.HHH_glszm_ZoneVariance | 0.997(0.996-0.998) | 0.997(0.996-0.998) |
| wavelet.LHH_glszm_SizeZoneNonUniformity | 0.985(0.979-0.990) | 0.988(0.983-0.992) |
| X3dresnet.feature1021 | 0.969(0.956-0.979) | 0.969(0.956-0.979) |
| log.sigma.1.0.mm.3D_glszm_GrayLevelNonUniformityNormalized | 0.974(0.962-0.982) | 0.976(0.965-0.983) |
| log.sigma.5.0.mm.3D_firstorder_Kurtosis | 0.998(0.997-0.999) | 0.998(0.997-0.999) |
| wavelet.LHL_glcm_InverseVariance | 0.998(0.997-0.999) | 0.998(0.997-0.999) |
| log.sigma.3.0.mm.3D_glszm_SizeZoneNonUniformityNormalized | 0.979(0.970-0.985) | 0.982(0.974-0.987) |
| wavelet.LHH_glszm_GrayLevelNonUniformityNormalized | 0.904(0.864-0.932) | 0.929(0.900-0.950) |
| wavelet.HLH_firstorder_Skewness | 0.998(0.997-0.999) | 0.998(0.997-0.999) |
| log.sigma.2.0.mm.3D_glszm_SmallAreaLowGrayLevelEmphasis | 0.985(0.978-0.990) | 0.986(0.980-0.990) |
| wavelet.HLH_glrlm_ShortRunEmphasis | 0.995(0.992-0.996) | 0.994(0.992-1.000) |
| log.sigma.1.0.mm.3D_glszm_SmallAreaEmphasis | 0.951(0.929-0.966) | 0.952(0.931-0.967) |
| log.sigma.1.0.mm.3D_glszm_SizeZoneNonUniformityNormalized | 0.968(0.953-0.978) | 0.974(0.962-0.982) |
| wavelet.HHH_glszm_GrayLevelNonUniformityNormalized | 0.986(0.981-0.991) | 0.986(0.981-0.991) |
| wavelet.HHL_glszm_SmallAreaEmphasis | 0.996(0.994-0.997) | 0.996(0.994-0.997) |
| log.sigma.4.0.mm.3D_glszm_SizeZoneNonUniformityNormalized | 0.981(0.973-0.987) | 0.989(0.985-0.992) |
| wavelet.HHL_glcm_InverseVariance | 0.991(0.987-0.994) | 0.987(0.982-0.991) |
| wavelet.LLH_glrlm_RunPercentage | 0.984(0.978-0.989) | 0.983(0.976-0.988) |
| wavelet.HLH_glcm_JointEntropy | 0.996(0.994-0.997) | 0.996(0.994-0.997) |
| X3dresnet.feature1041 | 0.948(0.926-0.964) | 0.948(0.925-0.963) |
| wavelet.LLH_glszm_SmallAreaLowGrayLevelEmphasis | 0.949(0.927-0.964) | 0.951(0.931-0.966) |
| wavelet.HHH_glcm_Imc2 | 0.996(0.994-0.997) | 0.995(0.992-0.996) |
| wavelet.LLH_firstorder_Kurtosis | 0.998(0.997-0.999) | 0.998(0.997-0.999) |
| wavelet.HLL_gldm_LargeDependenceLowGrayLevelEmphasis | 0.979(0.970-0.986) | 0.979(0.970-0.986) |
| log.sigma.4.0.mm.3D_glszm_SmallAreaLowGrayLevelEmphasis | 0.979(0.970-0.985) | 0.985(0.978-0.989) |
| wavelet.LHL_gldm_DependenceVariance | 0.998(0.996-0.999) | 0.998(0.997-0.999) |
| wavelet.LLL_firstorder_InterquartileRange | 0.886(0.836-0.920) | 0.883(0.833-0.919) |

**Supplementary figure S1** Feature Screening Related Processes


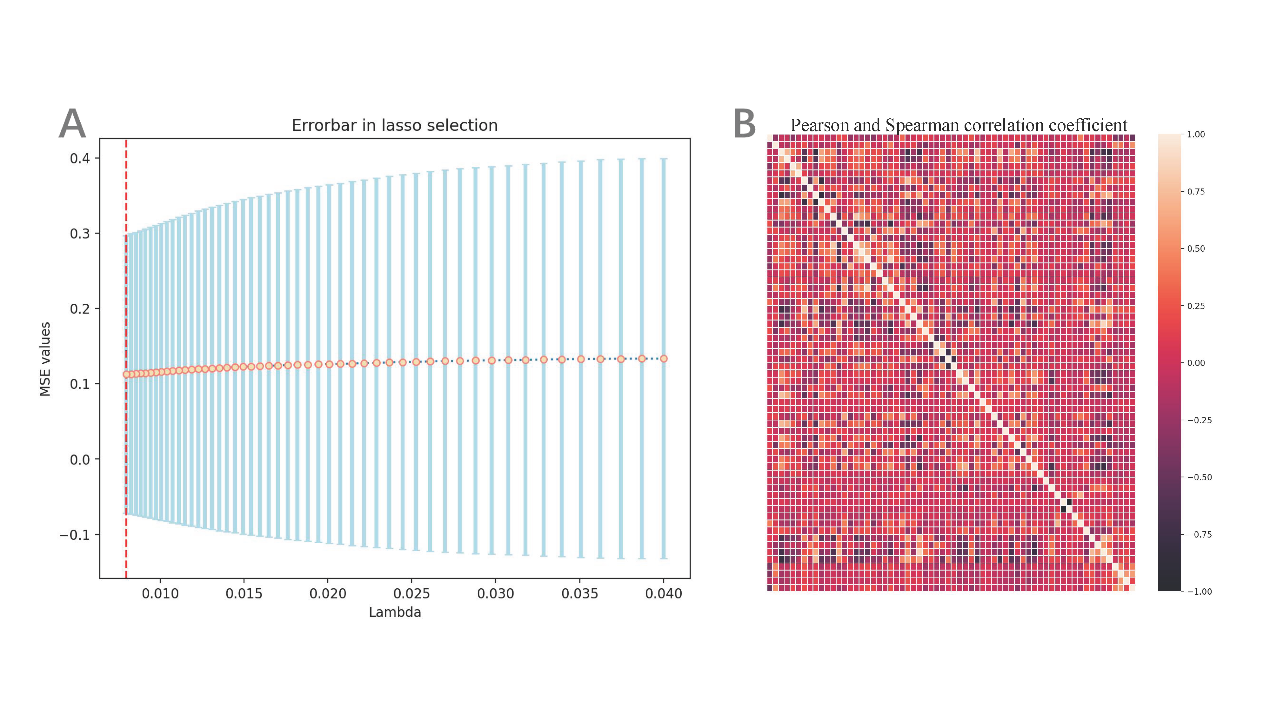


(A: The error bar plot in lasso selection; B: Pearson and Spearman correlation coefficients, all feature correlation coefficients are less than the absolute value of 0.9)

**Supplementary figure S2** The detailed 3D-cropbox workflow


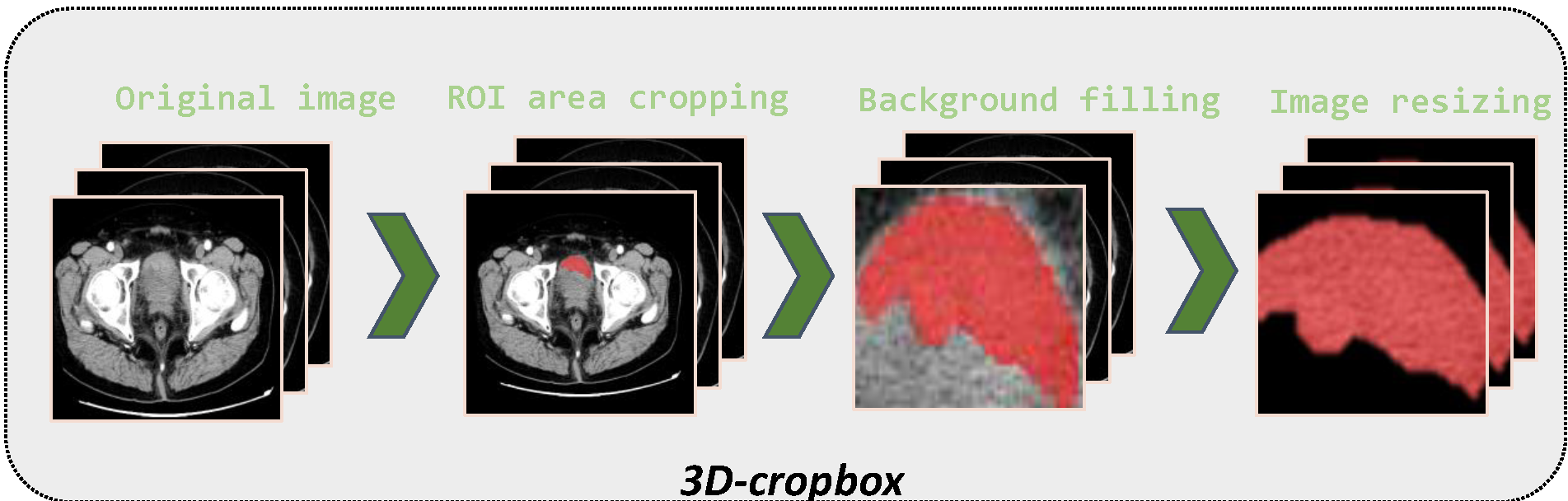


**Supplementary figure S3** Calibration curve in the training cohort


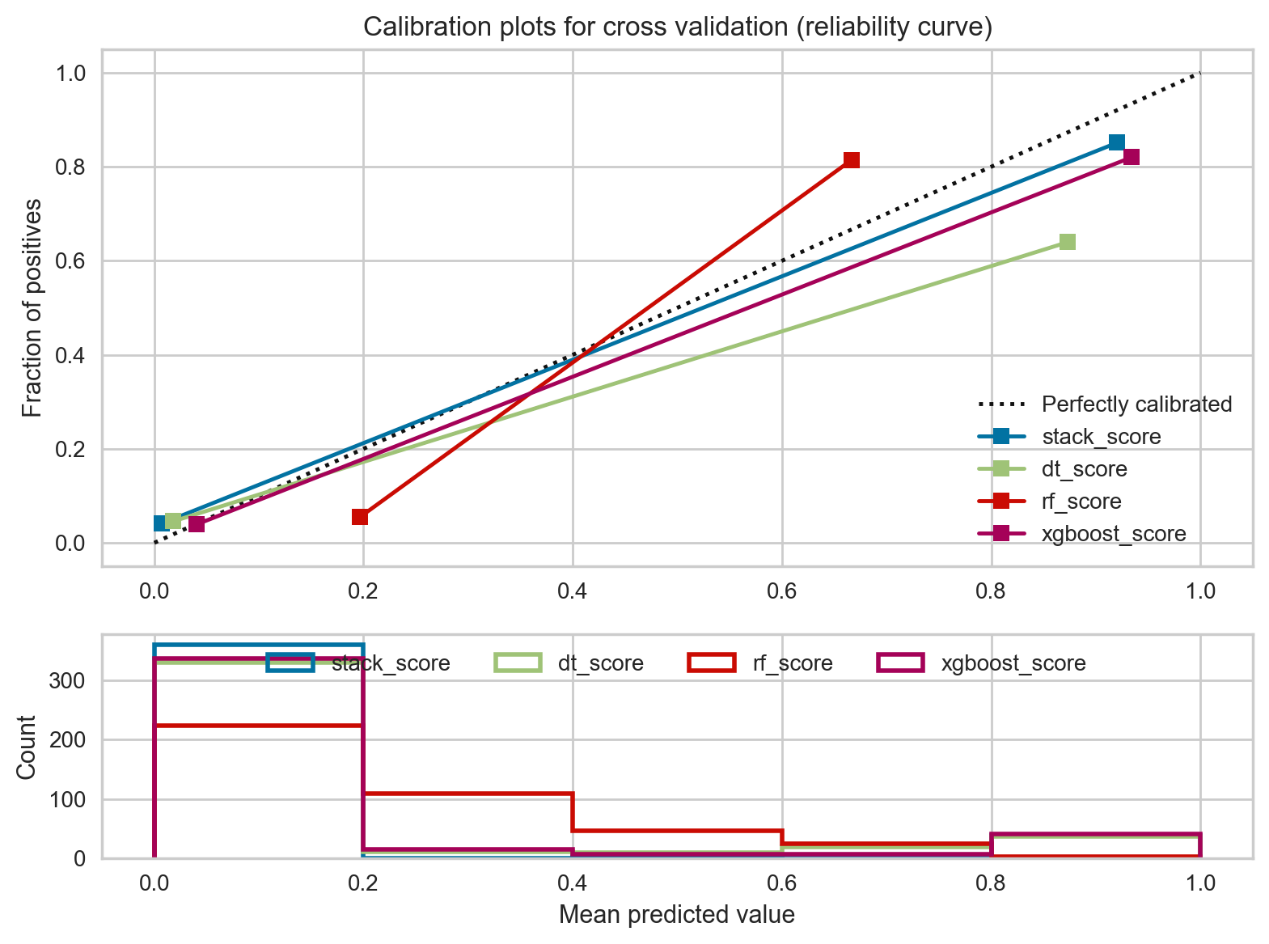


Legend: The black dashed line represents the ideal prediction curve. As the prediction curve of machine learning model approaches the dashed line, the model becomes more accurate.

**Supplementary figure S4** Calibration curve in the external validation cohort


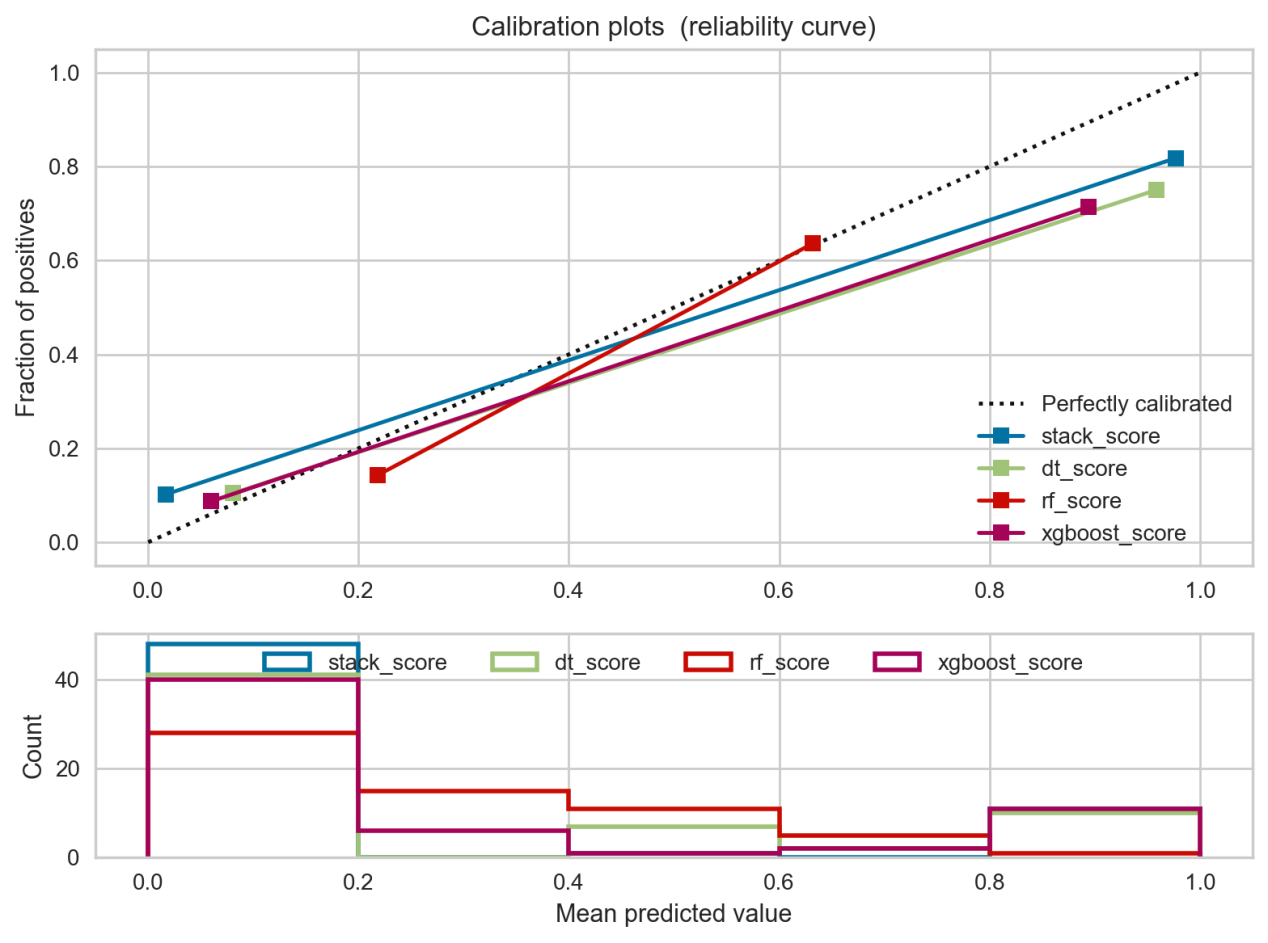


Legend: The black dashed line represents the ideal prediction curve. As the prediction curve of machine learning model approaches the dashed line, the model becomes more accurate.

**Supplementary figure S5** An example of histopathological image with corresponding CT images

**
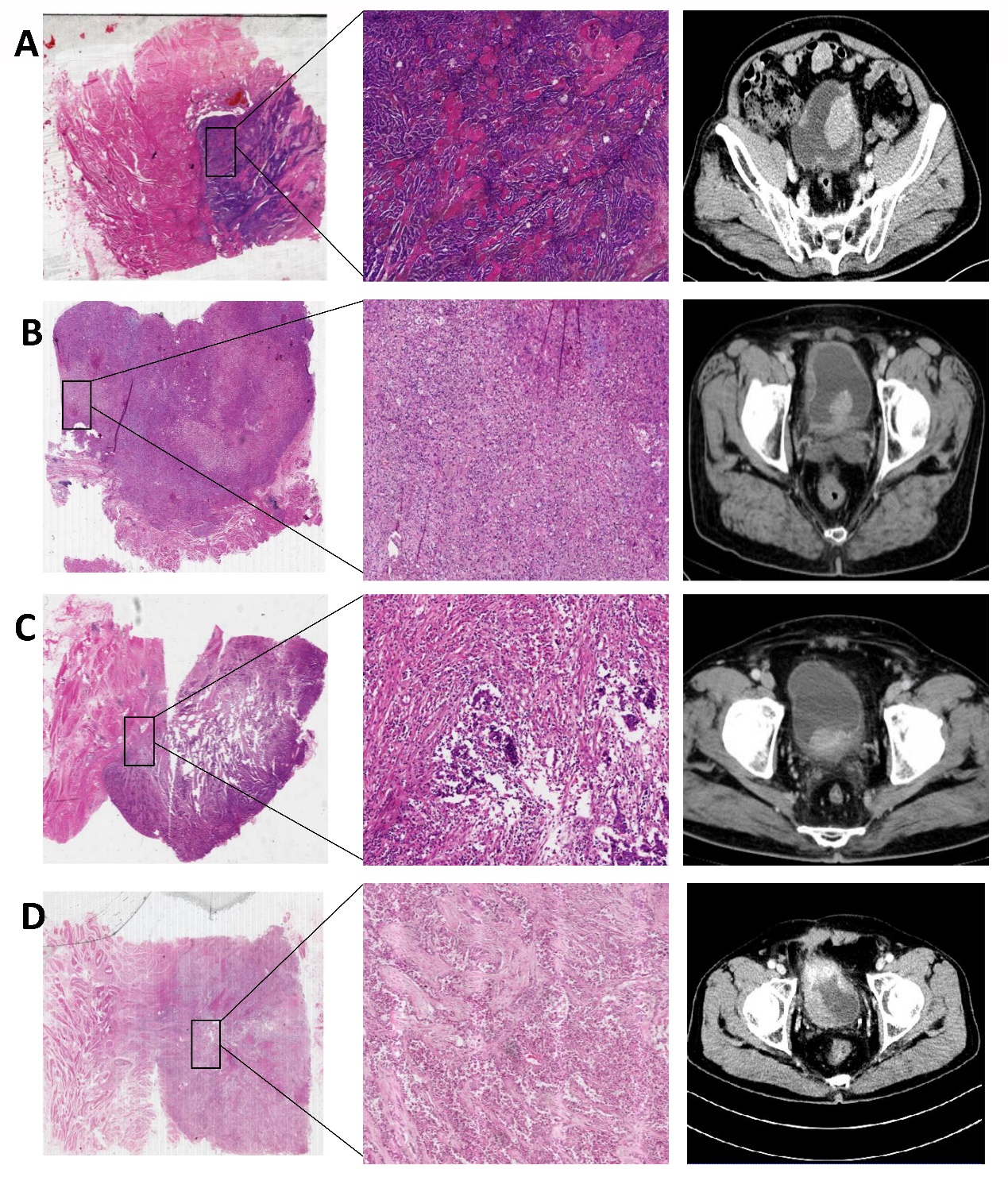
Figure A**: High-grade invasive urothelial carcinoma with squamous differentiation, invading the deep muscular layer. **Figure B**: Focal squamous differentiation with micropapillary urothelial carcinoma components (approximately 20%). **Figure C**: Invasive high-grade urothelial carcinoma with focal neuroendocrine differentiation. **Figure D**: Invasive high-grade urothelial carcinoma with partial glandular differentiation.

Supplementary figure S6 The confusion matrix for cross-validation set

*
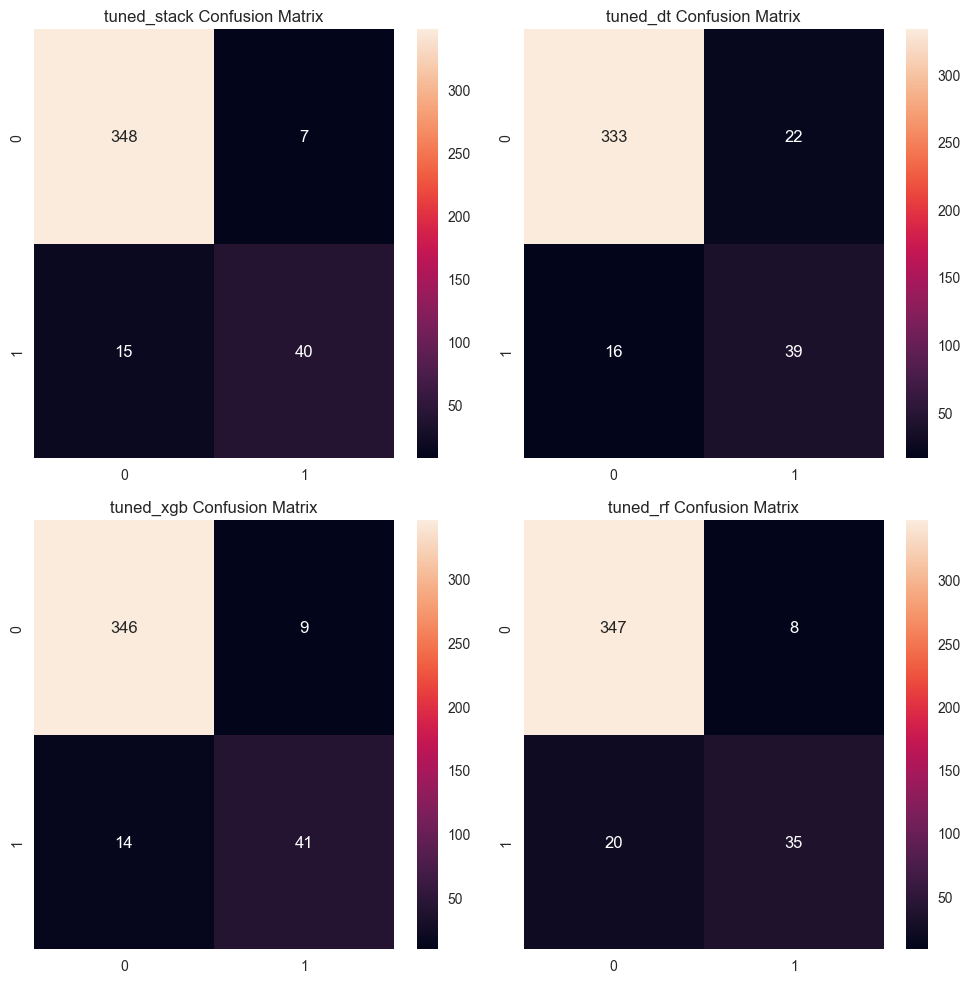
*

Supplementary figure S7 The confusion matrix for external validation set


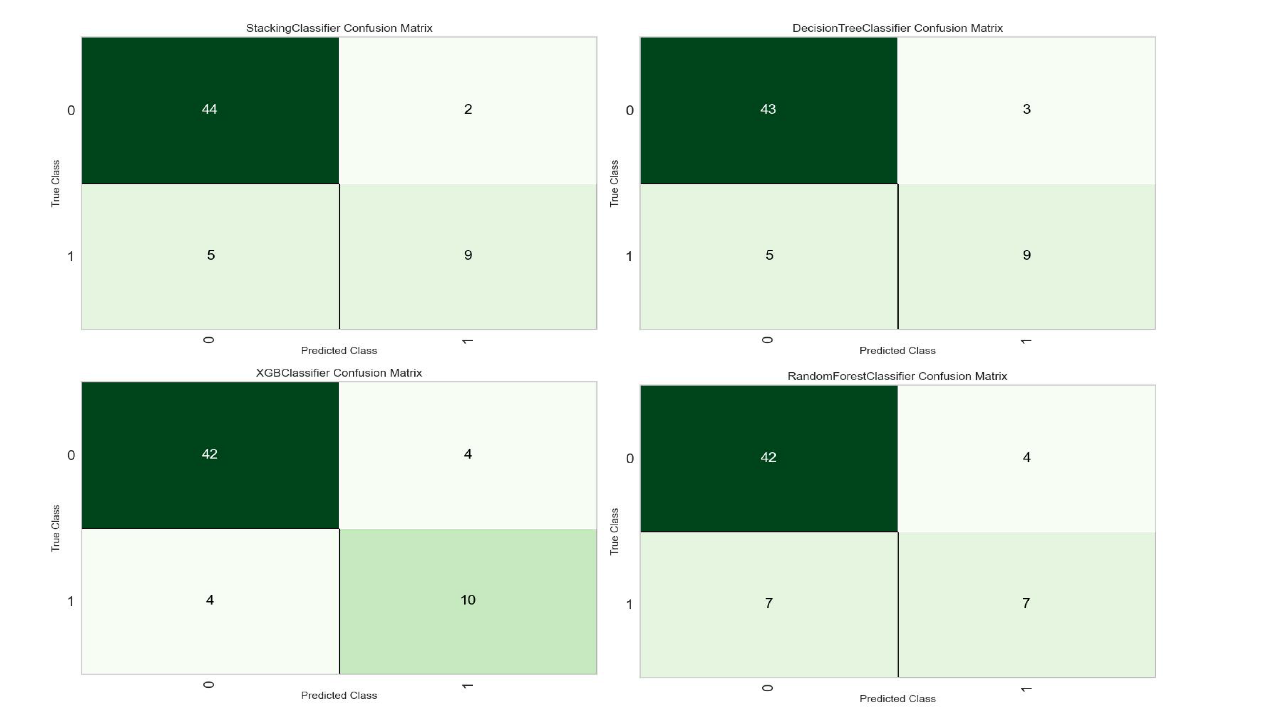


Supplementary figure S8 Decision curve analysis for four machine learning systems in external validation dataset


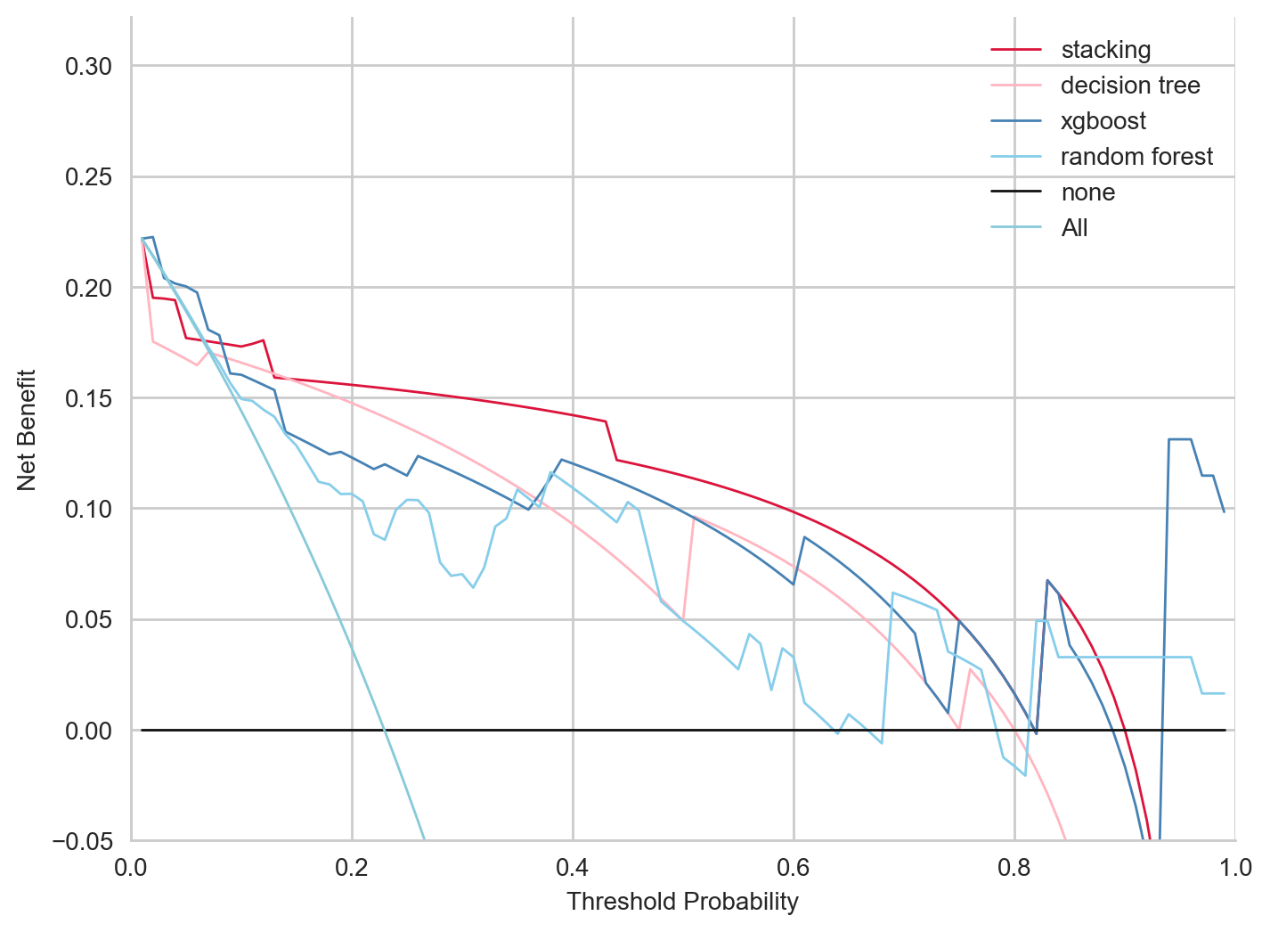


Legend: The net benefit is represented on the y-axis and corresponding threshold probability is represented on the x-axis. The stacking ensemble system is represented by the red line

Supplementary figure S9 heatmap of the correlation coefficients


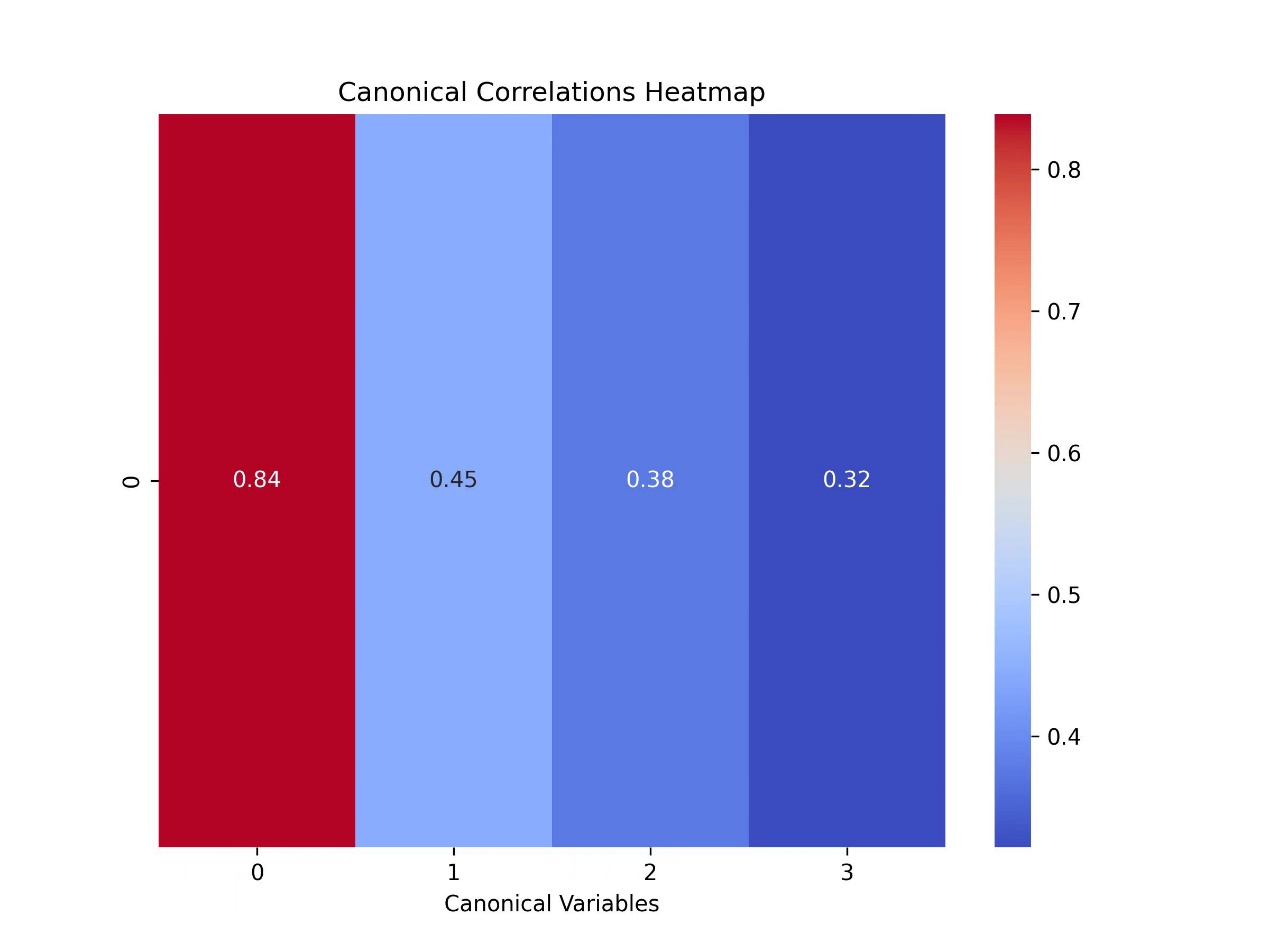


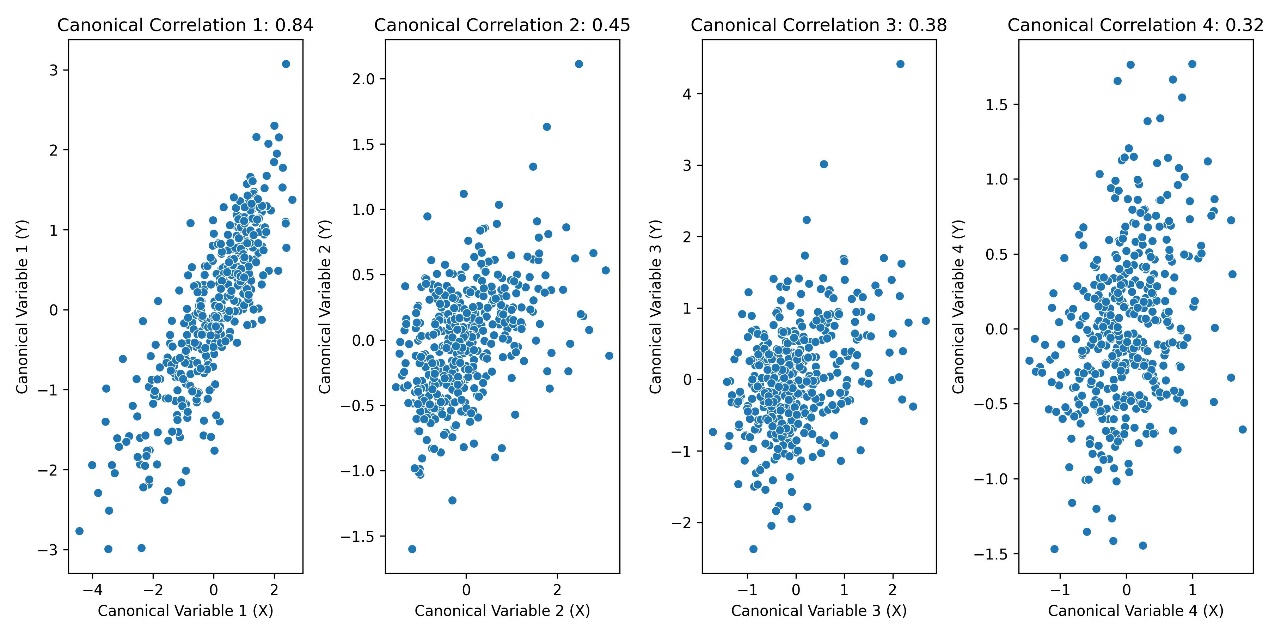

Supplement: Supplementary file 1 [file SupplementaryFile1.docx]
